# The cyclical cascade of HIV care: Temporal care engagement trends within a population-wide cohort

**DOI:** 10.1371/journal.pmed.1004407

**Published:** 2024-05-10

**Authors:** Jonathan Euvrard, Venessa Timmerman, Claire Marriott Keene, Florence Phelanyane, Alexa Heekes, Brian D. Rice, Anna Grimsrud, Peter Ehrenkranz, Andrew Boulle

**Affiliations:** 1 School of Public Health, University of Cape Town, Cape Town, South Africa; 2 Department of Health, Provincial Government of the Western Cape, Cape Town, South Africa; 3 Centre for Tropical Medicine and Global Health, University of Oxford, Oxford, United Kingdom; 4 School of Medicine and Population Health, University of Sheffield, Sheffield, United Kingdom; 5 Faculty of Public Health and Policy, London School of Hygiene and Tropical Medicine, London, United Kingdom; 6 HIV Programmes and Advocacy, IAS–the International AIDS Society, Cape Town, South Africa; 7 Global Health, Bill & Melinda Gates Foundation, Seattle, Washington State, United States of America

## Abstract

**Background:**

The traditional HIV treatment cascade aims to visualise the journey of each person living with HIV from diagnosis, through initiation on antiretroviral therapy (ART) to treatment success, represented by virological suppression. This representation has been a pivotal tool in highlighting and quantifying sequential gaps along the care continuum. There is longstanding recognition, however, that this may oversimplify the complexity of real-world engagement with HIV services in settings with mature high-burden HIV epidemics. A complementary “cyclical” cascade has been proposed to represent the processes of disengagement at different points on the care continuum, with multiple pathways to re-engagement, although the feasibility of implementing this at scale has been uncertain. This study aimed to populate, refine, and explore the utility of a cyclical representation of the HIV cascade, using routine data from a high-burden HIV setting.

**Methods and findings:**

This observational cohort study leveraged person-level data on all people living with HIV in the Western Cape (WC), South Africa, who accessed public health services in the 2 years prior to 31 December 2023. Programme data from disease registers were complemented by data from pharmacy and laboratory systems. At study closure, 494 370 people were included, constituting 93% of those of those estimated to be living with HIV in the province, of whom 355 104 were on ART. Substantial disengagement from HIV care was evident at every point on the cascade. Early treatment emerged as a period of higher risk of disengagement, but it did not account for the majority of disengagement. Almost all those currently disengaged had prior experience of treatment. While re-engagement was also common, overall treatment coverage had increased slowly over 5 years. The transition to dolutegravir-based regimens was dramatic with good virological outcomes for those in care, notwithstanding a clearly discernible impact of the Coronavirus Disease 2019 (COVID-19) pandemic on viral load (VL) testing. People currently engaged and disengaged in care are similar with respect to age and gender. Those who died or disengaged recently were previously distributed across a range of cascade statuses, and a substantial proportion of those newly initiating and re-initiating treatment were no longer on treatment 6 months later. The main limitation of this study was incomplete evidence of HIV testing, linkage to HIV-specific services, and out-of-facility mortality.

**Conclusions:**

Using routine data, it was possible to populate and automate a cyclical cascade of HIV care that continuously captured the nonlinear care journeys of individuals living with HIV. In this generalised mature HIV epidemic, most people are treatment experienced. Disengagement is common and occurs at various points along the cascade, making it challenging to identify high-impact intervention opportunities. While historical HIV cascades remain valuable for target setting and service monitoring, they can be complemented with insights from more detailed cyclical cascades.

## Abstract 2

Jonathan Euvrard and colleagues leverage routinely collected data from a high-burden HIV setting to demonstrate the feasibility of populating a cyclical HIV care cascade as part of routine service provision and to identify care gaps and temporal trends and changes to cascade statuses.

## Author summary

**Why was this study done?:**

- While there had been broad enthusiasm for cyclical representations of the HIV care cascade, the feasibility and utility of populating such representations using routine data in high-burden settings like South Africa are unknown.

**What did the researchers do and find?:**

- The researchers used routine person-level data from 494 370 people who recently accessed public health services in the Western Cape (WC), South Africa, to adapt and populate a cyclical cascade of HIV care.
- The study uncovered complex patterns of engagement, disengagement, and re-engagement at various cascade points. Early treatment emerged as a period of higher risk of disengagement, but it did not account for the majority of disengagement.
- Trends over time demonstrated the shift among those starting ART from first-ever HIV engagement to re-engagement and service disruptions resulting from the Coronavirus Disease 2019 (COVID-19) pandemic.

**What do these findings mean?:**

- With an appropriate data consolidation environment within the health services, it is readily feasible to continuously populate a cyclical representation of the HIV cascade of care using routine administrative and clinical data.
- The findings provide insights into the complex nature of engagement with HIV care. The identification of disengagement at multiple points on the care journey suggests the need for scalable, inclusive interventions.
- While historical cascades remain important tools, the cyclical cascade offers a complementary understanding of engagement patterns, contributing to ongoing efforts to refine public health strategies and improve HIV care outcomes.
- The main limitation of this study was incomplete evidence of HIV testing, linkage to HIV-specific services and out-of-facility mortality.

## Introduction

In earlier years of the HIV response, global targets focused on expanding treatment coverage for people living with HIV who were eligible [1–4]. With the introduction of universal test and treat (UTT) in 2015 [5], global targets evolved to encompass population-wide goals for testing, treatment, and virologic suppression [6,7]. A linear cascade emerged representing sequential gaps along a care continuum from HIV testing through to antiretroviral therapy (ART) initiation and treatment success (virologic suppression) [4]. This cascade paradigm has proven a valuable operational and advocacy tool in HIV service delivery [3,8]. Even in the absence of digitised health records, various methods have been employed to estimate coverage at each cascade step utilising a range of surveillance and other data sources [9–11].

There is longstanding recognition that the sequential nature of the standard cascade representation may oversimplify the complexity of real-world engagement with HIV services [12,13]. It conveys a false sense of linearity to care processes, which has the potential to emphasise initial case identification and first-time treatment initiation at the expense of retention in care of those already initiated, whereas both are crucial to achieving population-level outcomes. It could also result in an underappreciation of the cycling in and out of care that is inevitable in long-term chronic disease programmes [14–16].

A complementary “cyclical” cascade has been proposed to represent the processes of disengagement at different points on the care continuum, with multiple pathways to re-engagement [14]. Extending the cascade representation offers potential benefits: it could provide a deeper understanding of the challenges people living with HIV encounter during their treatment journey. This understanding may support the development of more targeted interventions to improve HIV care outcomes. While not everyone initiating ART will experience treatment interruptions, it is common enough that programmes seeking to decrease morbidity, mortality, and transmission of new infections will need to proactively anticipate and address disengagement [17,18].

Advocates for cyclical representations of the HIV cascade have highlighted the challenges of compiling robust information to populate such representations [14,19]. This is especially the case in high HIV-burden settings with multiple information system challenges, including weak or nascent electronic health information systems, absence of unique health identifiers, and the unreliability of self-reported data about prior care [19,20]. Dedicated case-based surveillance (CBS) initiatives have been proposed to assemble linked individuated HIV data [21].

By mid-2023, it was estimated that there were almost 8 million people living with HIV in South Africa, of whom an estimated 530 089 people were living with HIV in the Western Cape (WC) province [22,23]. While HIV prevalence is lower than some other provinces, it remains a high HIV-burden setting (>7% prevalence). There is a robust civil registration system in South Africa. However, civil identifiers are not always shared by patients with the health services. The WC was a relatively early adopter of a unique health identifier (UID) starting with the implementation of a shared hospital information system in 2001, which was progressively extended to all 52 hospitals over 2 decades [24]. From 2007, the UID was leveraged and shared by primary healthcare patient registration systems in 354 clinics, until the UID was implemented across all fixed health facilities. In 2015, the WC formally initiated a consolidated data environment for person-level health data, the Provincial Health Data Centre (PHDC), which has sought to enumerate cohorts for people with priority health conditions such as HIV, tuberculosis, pregnancy, and diabetes [25–27].

This paper aims to use real data from this high-burden HIV setting to populate the HIV cyclical cascade, demonstrate the feasibility of doing this as part of routine service provision, and identify care gaps by characterising the distribution of people living with HIV on the cascade, temporal trends in cascade statuses, and movements between them.

## Methods

### Study design and population

We conducted a reflective review of a health information system implementation initiative and a cohort study assembled from routine health data. The cohort is enumerated continuously using existing routine data collected by public health services for the purpose of providing healthcare to people living with HIV and for health service monitoring and evaluation. The study population for the presented illustrative cascade included all people living with HIV in the WC who had accessed public health services in the 2 years prior to 31 December 2023 or prior to the operative reporting date for temporal trend analyses.

### Ethics

Approval for this analysis was received from the Human Research Ethics Committee at the University of Cape Town (379/2021) and the Provincial Health Research Committee (WC_202007_043).

### The HIV programme and associated information systems

Starting in 2001, ART has been provided free-of-charge by public health clinics to people living with HIV in the WC province, with eligibility criteria evolving according to international guidance, culminating in UTT from September 2016 [28]. The high uptake of public services among people living with HIV and the availability of electronic laboratory results, medicine dispensing records, and clinical visits linked by UID presents a unique opportunity to follow a large cohort of people living with HIV through the cyclical cascade over time.

Testing for HIV in public primary healthcare settings is usually performed using a rapid point-of-care test followed, if positive, by another rapid confirmatory test. The results of these tests have started to be captured electronically at person-level, but currently coverage is estimated to be around 20%. Previously, it was assumed that patients with positive test results would be captured to an electronic HIV register like TIER.Net or discernible through follow-up laboratory tests (see below), while confirmation of testing negative was more difficult to ascertain digitally at person-level [29].

CD4+ T-lymphocyte cell count (CD4) and viral load (VL) tests done in the WC are performed by central laboratories, with digitised results available to the PHDC [19,30]. CD4 tests are no longer required to assess ART eligibility but are still recommended to assess immune system function in those initiating ART for the first time or restarting ART after a treatment interruption. In the absence of a digitised HIV test result or HIV register entry, the CD4 result from the laboratory may be the first digital evidence of a person’s HIV–positive status. VL tests have been recommended to confirm response to treatment at 6 months after starting ART, but in the WC have usually been done at 4 months. Thereafter, VL tests are recommended 12 months after starting ART and then annually in patients who remain virologically suppressed, while patients with viraemia are eligible for earlier repeat testing following adherence interventions.

ART starts, restarts, visits, and regimens are captured using the same UID to one of 3 HIV register systems, namely TIER.Net, Prehmis, or PHCIS (see S1 Appendix for more information) [29]. In all hospitals and many primary care facilities, ART dispensing is also captured to pharmacy information systems. If an ART start or restart event is not captured to the register systems, it may still be inferred from dispensing evidence in the pharmacy data. Almost all ART is provided in primary care, mostly by nurses, with visits typically 1 or 2 months apart. There are a number of repeat prescription collection strategies which do not involve clinical consultations [31–37], which are usually facilitated by pre-packaged medicine parcels, with digitised information on delivery and collection in most cases.

Regimens have evolved in line with global guidance [38]. Starting in 2013, a fixed-dose ART regimen containing a non-nucleoside analogue reverse transcriptase inhibitor (NNRTI) simplified prescribing and reduced pill burden. Since 2020, this has gradually been replaced with a fixed-dose regimen containing an integrase strand transfer inhibitor (INSTI). It is hoped that rollout of the new regimen will lead to increased levels of viral suppression [39].

The Single Patient Viewer was developed as a web-based interface to provide a longitudinally integrated health record based on the data held in the PHDC for use by clinicians, and later became the reporting portal for reports based on disease cascades, available either as actionable line lists or interactive reports [25]. This is the target platform for routine presentation and use of the new cyclical cascade of HIV care, by clinicians and health programme managers.

### Data management and analysis

This study is reported as per the Strengthening the Reporting of Observational Studies in Epidemiology (STROBE) guideline (S1 STROBE Checklist). Comprehensive details about the data management within the PHDC, allowing for the continuous curation and updating of the HIV cohort [40], can be found in the supplementary Technical Appendix, covering data transfer from source systems, data curation and alignment with common data structures, the implementation of phenotype algorithms for cohort inclusion, and the data processing necessary to flexibly estimate current and prior cascade statuses.

The analyses in this paper are based on the operational “HIV cascade report,” which is accessible online by health service staff. This is a detailed interactive report based off the cyclical cascade which is updated daily and accessible to clinicians and managers. It contains the visuals presented in this manuscript and many more interactive tools for exploring the HIV population, filterable by service area. Practical adaptations to the steps of the originally proposed cyclical cascade are described, alongside data processing adjustments to enable dynamic population of the cyclical cascade for different historical dates.

The presented analyses of the populated cascade follow the structure of the operational report, which describes: the characteristics of patients on the cascade, the distribution of patients on the cascade at a selected point in time, temporal trends in the absolute and relative numbers of patients in cascade statuses, and individual patient movements between cascade statuses over time.

Patient characteristics are described by sex, age group, VL suppression status (not yet eligible for testing, suppressed, elevated, not evaluated in the preceding 15 months), and CD4 count at ART start (categorised as below 200, 200 to 349, 350 to 499, or 500 and above cells/μl).

Temporal trends (presented quarterly) are described for CD4 count distribution (by the above categories and median) among people starting ART, the number of people starting ART among truly ART-naïve patients versus patients restarting ART after an interruption, the total number of people on ART by VL suppression status, and the distribution of patients by anchor drug class (i.e., NNRTI, PI, or INSTI). The Thembisa mathematical model of the South African HIV epidemic was used to provide a comparison of the total number of included patients to the best estimate of the provincial HIV population [41].

Illustrative examples of prior and subsequent cascade statuses focus on statuses 6 months prior to disengagement or death, and outcomes 6 months after being on ART. All outputs are reported or visualised as patient totals or proportions, and are presented without confidence intervals or hypothesis testing, given the routine data system focus of the study, and that the entire population is included without sampling.

### Linear and cyclical HIV treatment cascades

**Fig 1:**
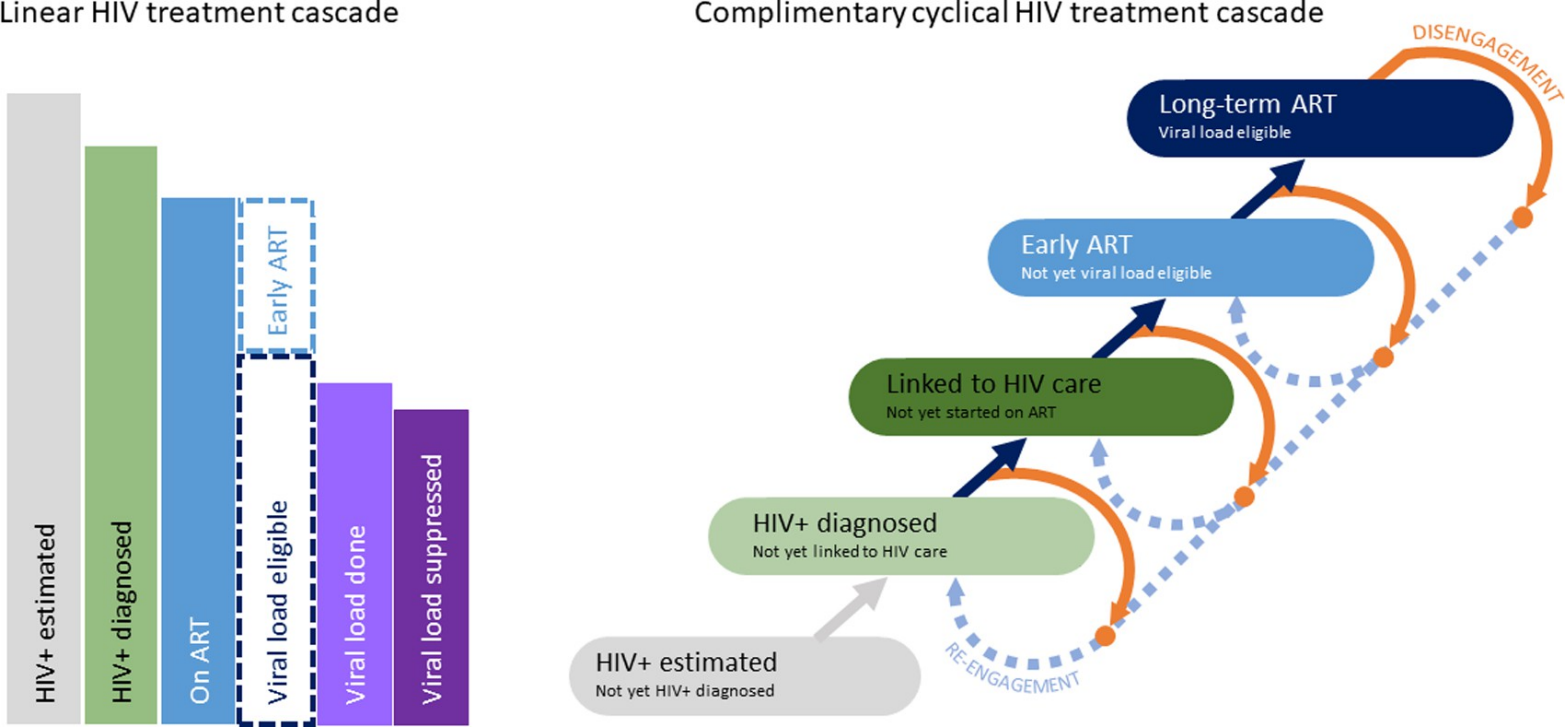
Linear and cyclical HIV treatment cascades: statuses with dotted borders are not typically representated on linear cascades, but disaggregation is facilitated when constructing the linear cascade from mutually exclusive cyclical cascade statuses. ART, antiretroviral therapy.

Both representations of the HIV treatment cascade shown in Fig 1 visualise the journey for those living with HIV from not knowing their HIV status to diagnosis, treatment initiation, and ultimately achieving viral suppression. One of the strengths of the linear cascade on the left is that it represents the proportions of people who reach different milestones on their journey to successful treatment on ART, providing simple and powerful summative metrics. Each status moving from left to right is a subset of the previous status. The cyclical cascade on the right divides the in-care population into mutually exclusive statuses, and adds disengagement statuses for those out of care. It seeks to track patients at each point in time as they move between statuses following a variety of pathways, and explicitly differentiates those who have previously been in care from those newly entering care (HIV or ART care). Each status in the linear cascade can be reconstructed through the selective combining of mutually exclusive statuses on the cyclical cascade. Statuses on the cyclical cascade in turn can be further disaggregated, such as dividing those on long-term ART into subgroups based on VL testing and outcomes within a preceding time window.

### Cyclical cascade adaptations

The initial intent was to populate the cyclical cascade exactly as proposed by Ekhrenkranz and colleagues [14]. The proposed cyclical cascade had categories for patients diagnosed with HIV, linked to HIV care, initiated on ART, retained on ART early for 6 months or less, and retained on long-term ART, with the possibility of disengaging from any of these categories, and re-engaging directly back to care or through repeat HIV testing. Based on the data available, some adaptations and expansions were applied to the previously proposed cascade statuses (Fig 1), and specific definitions for each cascade status appropriate to the WC context were refined (Table 1).

**Table 1:** Definitions of the statuses on the cyclical and linear HIV cascades.

Status on the cascade	Definition
Cyclical cascade	
HIV+ modelled	Thembisa model estimate of those living with HIV in the province.
HIV+ diagnosed or HIV+ rediagnosed^a^	Known with HIV based on digitised person-level evidence, without evidence of having died or migrated out of the province.
Linked to HIV care or Relinked to HIV care^b^	Linked to HIV care based on digitised evidence of preparation for ART initiation, not yet started or restarted on ART.
Early ART or Early ART after restart^c^	Started/restarted on ART less than 6 months previously and therefore not yet eligible for viral load assessment.
Long-term ART or Long-term ART after restart^d^	Started/restarted ART at least 6 months previously and therefore expected to have had viral load assessed.
Disengaged^e^	Interrupted care for >90 days.
**Linear cascade**	
HIV+ modelled	Thembisa model estimate of those living with HIV in the province.
HIV+ identified	Known with HIV based on digitised person-level evidence.
On ART	On ART, based on current evidence of treatment in hand.
VL expected	On ART, at least 6 months since naïve ART initiation.
VL done	VL expected, and recent viral load done.
VL suppressed^f^	VL expected with recent viral load done <1 000 copies/ml.
VL undetectable	VL expected with recent viral load done <50 copies/ml.

^a^“HIV+ rediagnosed” describes a person who has disengaged at any point after initiating ART, i.e., was “Disengaged,” and then re-engaged by testing positive for HIV, but has not yet “Relinked to HIV care.”

^b^In the era of same-day initiation, it is still possible to disengage between diagnosis and starting/restarting ART.

^c^A threshold of 6 months for “Early ART” was used in the context of guidelines recommending a first viral load assessment at 4 months. As newer guidelines recommend earlier assessment, it may be appropriate to reduce this threshold.

^d^Long-term ART statuses are further disaggregated by viral suppression sub-statuses.

^e^An interruption threshold of 90 days was used in line with national reporting guidelines, i.e., >90 days with no visit for someone not previously on ART and >90 days without ART in hand for someone previously on ART.

^f^When calculating this as a percentage, the most appropriate denominator is VL expected, which consists of those people in the long-term ART status.

ART, antiretroviral therapy; VL, viral load.

**Fig 2:**
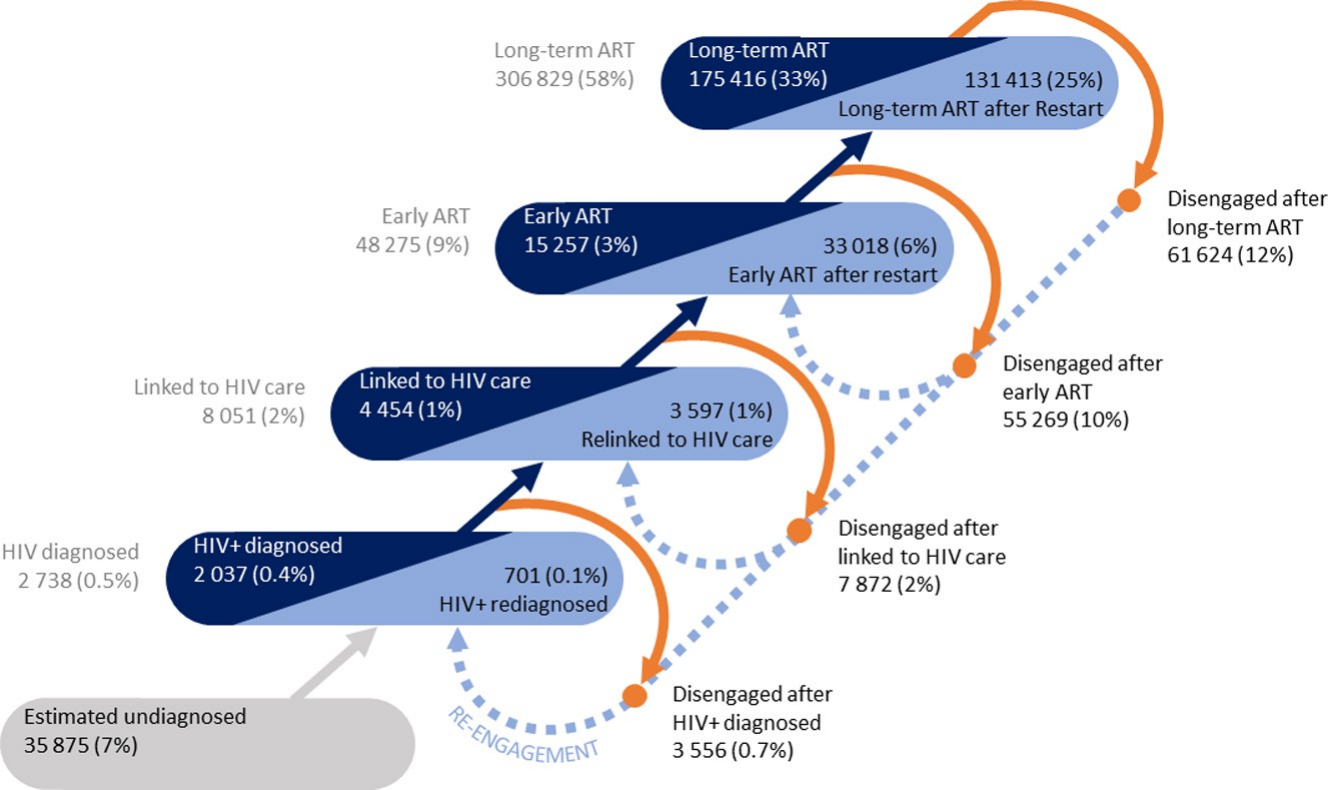
Absolute and proportional distribution of people living with HIV in the Western Cape, South Africa on 31 December 2023, across cyclical cascade statuses. ART, antiretroviral therapy.

The main adaptations to the proposed cascade were to ensure that each status (referred to as “stages” in the original proposal) represents a mutually exclusive category in which a patient could spend a variable amount of time, and each arrow represents a movement between statuses. This resulted in one less aggregated status (initiated/reinitiated ART) than originally proposed. As it was readily feasible with available data, the disaggregation by whether a patient had previously interrupted ART or not (Fig 2, light and dark blue, respectively) was maintained throughout the on-ART statuses in order to explore the utility of this differentiation.

Notable limitations to, or reasoning for, cascade status definitions include:

Both HIV+ diagnosis and HIV+ rediagnosis are under-ascertained due to a lack of digitisation of point-of-care HIV test results. Efforts are underway to digitise these events more comprehensively. In the interim, this remains an acknowledged shortcoming in the enumeration of these statuses.

While comprehensive data are collected on when and where a client has accessed public health services, a reliable indicator of what services were accessed is not currently available. According to guidelines, a person entering HIV care, either for the first time or after an interruption in care, should have a CD4 test performed. The presence of a CD4 result was used as a marker for entering or re-entering HIV care (“[Re]linked to HIV care” status). As above, this may also under-ascertain linkage to care if HIV-specific care was provided without repeating a CD4.

We considered that the originally proposed ART initiation or reinitiation status was actually an event indicating movement into the Early ART status, rather than being a status itself. Therefore, while it was included in the originally proposed cyclical cascade as a status, we opted to represent it as an arrow or movement between statuses, in this analysis. Individuals linked to HIV care were thereby separated from those linked and initiated on ART.

Early ART was defined as the first 6 months after ART initiation or reinitiation following disengagement. ART dispensing events are comprehensively captured, often in duplicate in both the HIV register and pharmacy information systems.

For consistency and to allow comparisons of people on ART who have or have not ever experienced an interruption, the distinction between ART after naïve initiation and ART after an interruption was maintained through Early ART and Long-term ART in the adapted cascade. The VL suppression categories described above were calculated as a mutually exclusive disaggregation to facilitate reporting on virologic outcomes.

There are 4 resulting disengagement statuses based on the corresponding in-care status prior to disengagement. Disengagement was defined as interrupting ART if on ART, or care if not on ART, for 90 days or more, in line with national guidelines [14,42]. A limitation is the inability to distinguish those who remain alive with HIV in the province from those with unascertained mortality or migration out of the province. Strategies to address this uncertainty are discussed below.

### Linear cascade adaptations

In order to report on the 95-95-95 cascade as part of the integrated cascade representation, the mutually exclusive “in care” statuses (HIV diagnosed/rediagnosed; Linked/Relinked to HIV care; Early ART/Early ART after restart; Long-term ART/Long-term ART after restart) were mapped to the relevant numerators and denominators of the linear cascade, resulting in some proposed refinements of the 95-95-95 definitions (Table 1).

Patients in the Early ART categories were excluded from both the numerator and denominator of the assessment of virologic suppression proportion. While it would be interesting to disaggregate the early ART group by VL status, in practice this is not yet feasible. According to provincial guidelines, a person who starts or restarts ART should have a VL assessment after 4 months on ART to assess treatment success (with an expectation that the timing of this might often be delayed). In the Early ART period before this first VL assessment, it is premature to further disaggregate into those not tested, suppressed, or viraemic.

When calculating summative indicators (those being well-managed as a proportion of all those known with or modelled to have HIV), patients on Early ART were added back into the numerator, alongside those on Long-term ART who were demonstrated to be virologically suppressed, to appropriately reflect successful management.

## Data processing adaptations

### Mutually exclusive statuses

We defined 13 mutually exclusive and exhaustive disaggregated cyclical cascade statuses and used parameterised branched algorithm logic to assign each person to a single status for any specified report date based on their last visit on or before that report date (see Technical Appendix). This was both computationally efficient on a large dataset and provided an accessible framework for troubleshooting and stakeholder engagement.

### Visit-centric data curation

To enable the branch logic to work for each patient for any report date, a data structure was required with a single row per person per service contact (see Table A in S1 Appendix). From this information, the last date on which the patient would have ART in hand is calculated, which is the key data point for determining retention in care. At the time of this service contact, the dates and values of the last CD4 and VL results are carried forward into the visit-level record to improve efficiency of retrieval for cascade branch logic and reporting.

### Pre-calculation of the entire cascade for historical dates

Originally, the intention was to provide a fully parameterised cross-sectional report. This approach would have allowed a user to provide a parameter, like the threshold for how many days without ART in hand at which a person is considered to have interrupted care, and any reporting date, and immediately see the cascade populated taking this threshold into consideration.

While the branch logic was highly efficient, the visit-level table contained over 40 million rows, and a sufficiently performant way to interactively process the dataset for multiple reporting dates was not readily available. Cascade statuses were therefore pre-calculated for a preselected set of historical dates and threshold definitions. This approach had the further benefit of enabling longitudinal or trend reporting and analysis. Limiting subsequent daily recalculation of visit histories and cascade statuses to only those patients for whom there was evidence of data changes, further limited the ongoing computational overhead of this paradigm.

## Results

Using the preselected default reporting parameters described above, 494 370 individuals known by public health services to be living with HIV in WC and who accessed services in the 2 years prior to 31 December 2023 were enumerated, representing 93% of estimated prevalence (Table 2). Of these, 354 421 people were on ART, which is 72% of those enumerated and 67% of those estimated to be living with HIV. Most people enumerated were treatment experienced (*n* = 477 638, 97%). The population included 336 466 females (68%). Median age was 39 years (IQR 32–47). The overwhelming majority (*n* = 409 767, 83%) of those enumerated were between 25 and 54 years of age. Among 305 699 people on Long-term ART, 253 229 (83%) had undetectable viral loads <50 copies/ml, and an additional 18 506 (6%) had suppressed viral loads <1 000 copies/ml.

**Table 2:** Characteristics of people living with HIV included in this study: shown for 31 December 2023.

Number of people included	494 370
Age (median, IQR)	39 (32–47)
Age category	
<10	4 964 (1%)
10–19	11 561 (2%)
20–29	72 418 (15%)
30–39	167 702 (34%)
40–49	149 946 (30%)
50–59	66 552 (13%)
≥60	21 227 (4%)
Female sex (*n*, %)	336 466 (68%)
Months since ART start (median, IQR)	81 (41–121)
Year of first ART start (median, IQR)	2017 (2013–2020)
Grouped year of first ART start	
<2009	34 826 (7%)
2009–2013	120 139 (25%)
2014–2018	149 566 (31%)
2019–2023	171 730 (36%)
Months since last ART start (median, IQR)	36 (15–82)
ART start CD4 category	
>500	72 472 (15%)
351–500	67 891(14%)
201–350	106 176 (22%)
<200	116 327 (24%)
Not available	130 815 (26%)
Last VL category (copies/ml)	
>1 000	11 761 (4%)
50–999	19 139 (6%)
<50	252 743 (83%)
Not available	22 056 (7%)
Last ART regimen	
TDF + 3TC/FTC + DTG	374 617 (77%)
TDF + 3TC/FTC + EFV	59 866 (13%)
ABC + 3TC/FTC + DTG	14 375 (3%)
ZDV + 3TC/FTC + DTG	8 818 (2%)
ZDV + 3TC/FTC + LPV/r	6 233 (1%)
Other	11 757 (2%)

ART, antiretroviral therapy; VL, viral load.

Cross-sectional distribution across the cyclical cascade

The starting point on both linear and cyclical cascades is the number of people estimated to be living with HIV in the WC. Unfortunately, this estimate is not yet available at a geographical or administrative level below that of the province as a whole.

The first proportion usually presented on the linear cascade is how many people living with HIV know their status. When we first calculated this proportion from the cyclical cascade, it was 115%. This was considered to be an overcount due to unascertained migration out of the province and under-ascertained deaths accumulating over 20+ years of the ART programme. In subsequent iterations, people not seen in the health services for 2 or more years were excluded, which resulted in the proportion of people living with HIV in the WC with known HIV+ status being calculated at 93%. This parameter was made dynamically adjustable to enable users to explore the impact of including or excluding patients based on when they were last active in the health services.

**Fig 3:**
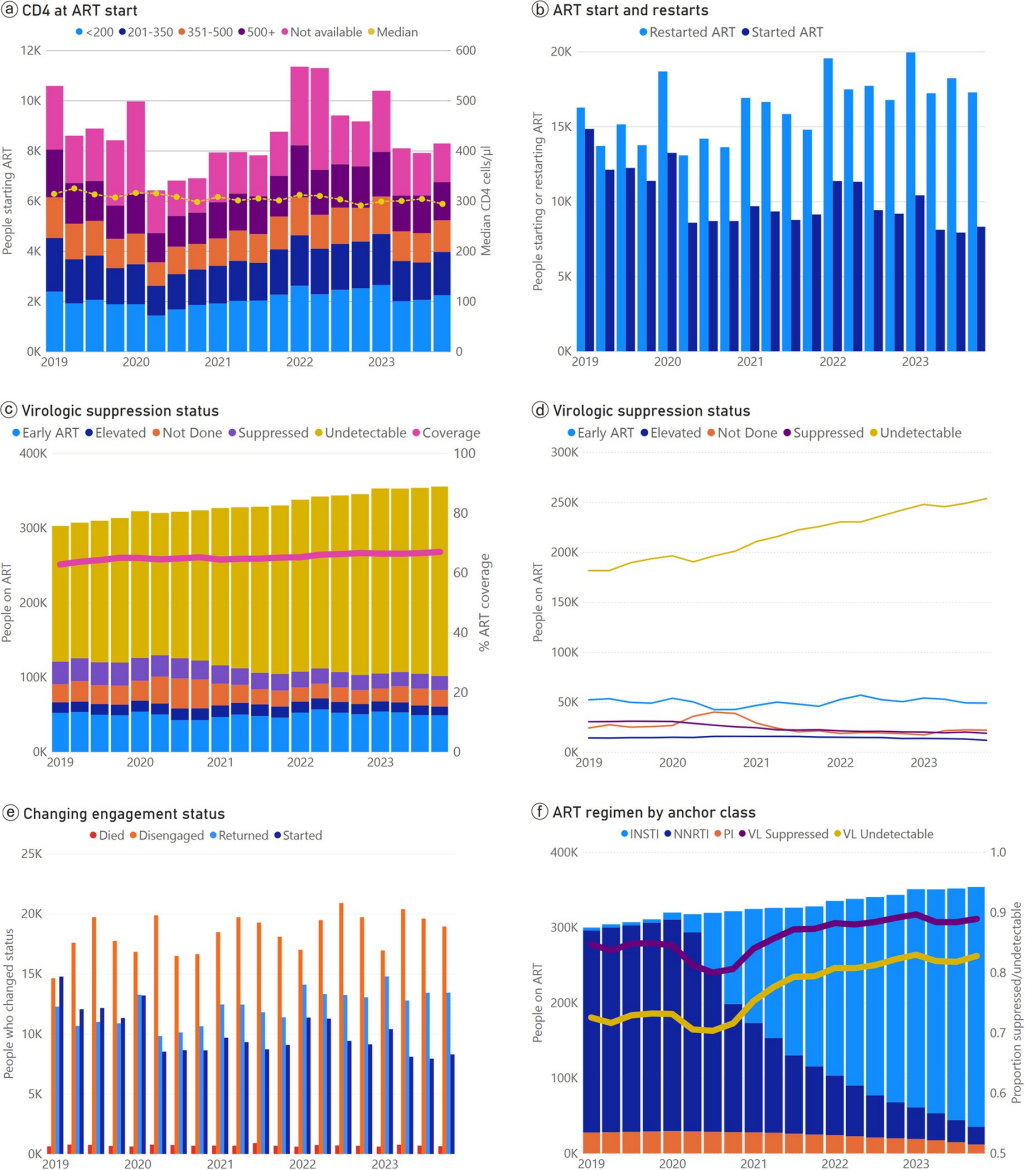
Temporal trends in the distribution of patients in and across cyclical cascade statuses, and in the characteristics of patients in selected cyclical cascade statuses, presented quarterly. ART, antiretroviral therapy; INSTI, integrase strand transfer inhibitor; NNRTI, non-nucleoside analogue reverse transcriptase inhibitor; VL, viral load.

An important insight that emerged when populating the cyclical cascade was that 48 722 (14%) of 354 421 people on ART in the WC were in the Early ART status after either starting or restarting ART (Figs 2 and 3C), with 33 394 (69%) on Early ART after restart compared to 15 328 (31%) on Early ART after naïve initiation.

Among 305 699 people on Long-term ART, 172 215 (56%) had never experienced an interruption in care since naïve initiation on ART. Among those on Long-term ART after restart, 72 802 (54%) had experienced only 1 interruption, followed by 24% and 12% who had experienced 2 or 3 interruptions, respectively.

The cyclical cascade clearly demonstrated that substantial numbers of people (26% of the cohort) are disengaged on any day, and that there are substantial numbers of disengagements from every engaged status on the cascade (Fig 2).

### Temporal trends in the numbers of people in and moving between cascade statuses

Plotting CD4 results at ART start categorically and by median, there was evidence of some seasonality, but overall the proportion starting ART with a CD4 <200 cells/μl has remained steady over the past 5 years (Fig 3A).

At the beginning of 2019, slightly more than half of those starting or restarting ART were restarting following at least 1 period of disengagement (Fig 3B). At the end of 2023, restarts accounted for over two thirds (*n* = 17 269, 68%) of all starts and restarts (25 564).

The total number of people on ART has slowly increased annually over the past 5 years (Fig 3C. The only time the total dropped quarter-on-quarter was as a result of the Coronavirus Disease 2019 (COVID-19) pandemic in 2020. While the total number of people with documented undetectable viral load dipped briefly in 2020 due to COVID-19, the number has been otherwise consistently increasing over the past 5 years (Fig 3D, yellow line). The absolute number of people on ART with an elevated VL >1 000 copies/ml has remained stable, but it has come to represent a smaller proportion of people on ART.

Between 15 000 and 20 000 people disengaged from care every quarter over the past 5 years, putting pressure on health services to start and restart many people to achieve the increase in overall ART coverage despite this disengagement (Fig 3E).

The change from NNRTI-based ART regimens to INSTI-based regimens is clearly reflected (Fig 3F). The dip in documented suppression due to COVID-19 and subsequent recovery is again noticeable, while the proportion of people with a confirmed undetectable viral load <50 copies/ml appears to have risen faster than the proportion suppressed at <1 000 copies/ml.

### Patterns of individual movement on the cascade

**Fig 4:**
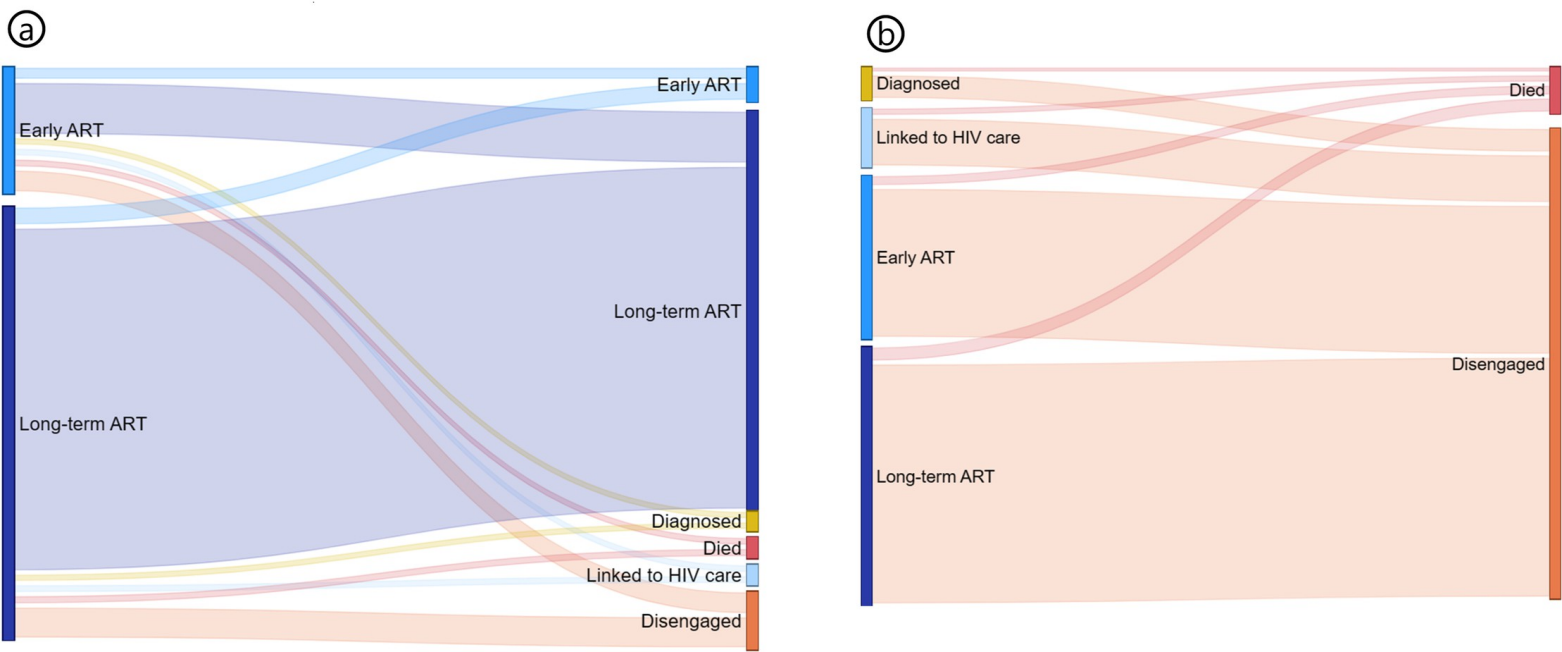
Movement between cyclical cascade statuses: (a) Current status among those on ART 6 months prior. (b) Status 6 months prior among those recently disengaged or who died during previous 6 months. Reported for 31 December 2023. ART, antiretroviral therapy.

Sankey diagrams were included on the cascade reports to provide a visual representation of movements of individuals between cascade statuses (Fig 4). For brevity, only 2 are shown here to demonstrate the dominant subsequent and antecedent statuses for patients on ART, and people who died or disengaged in the next or previous 6 months, respectively. Among those on Early ART, 69% progressed to the Long-term ART status over the next 6 months (Fig 4A). Among those in the Long-term ART status, 10% were no longer in the Long-term ART status 6 months later (Fig 4A). Looking at those newly disengaged, 53% were on Long-term ART 6 months prior and another 32% were on Early ART (Fig 4B). Of those who recently died, 31% had been on Long-term ART 6 months previously (Fig 4B).

### Characteristics of people engaged in or disengaged from care

**Fig 5:**
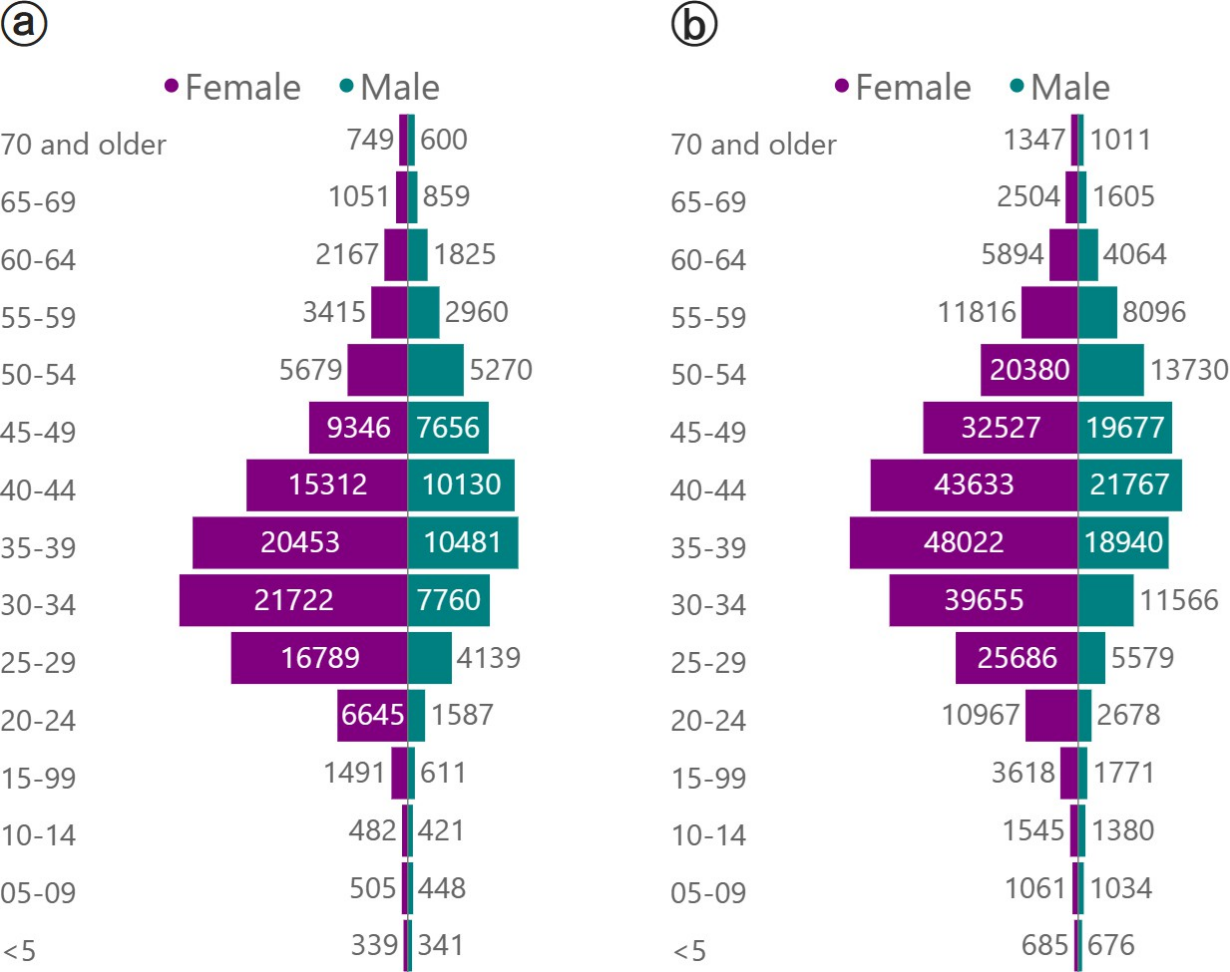
Comparing the age and sex distribution of (a) people engaged in care to (b) people disengaged from care. Report date: 31 December 2023.

Among males and females, the age profiles of those in care are broadly similar to those disengaged from care (Fig 5). Among 354 421 people on ART, 244 238 (69%) were female and median age was 40 years (IQR 33–47). The majority (83%) of those on ART were between 25 and 54 years of age. Comparatively, among 139 949 people not on ART but with service contact in the preceding 2 years, 92 228 (66%) were female and median age was 37 years (IQR 30–44). The majority (87%) of those not on ART were between 25 and 54 years of age.

## Discussion

We present a real-world attempt to populate a cyclical HIV cascade of care temporally and from the health services’ perspective, in which repeated engagement and reengagement in HIV care are explicit. The tools described in this paper demonstrate the feasibility of using routine individuated patient data in a high HIV-burden setting to flexibly represent the HIV cascade in different ways, and in a timely manner that can support service delivery and provide health service insights. Where data suffer from limitations or present challenges to interpretation, the resulting data representation can still be functional if there is transparency about the limitations, and if users can be guided on appropriate interpretation and use based on their specific requirements.

A number of challenges emerged when reflecting traditional ART coverage metrics. In the context of a mature ART programme with longstanding availability of data, it is not possible using routine data to accurately differentiate who, of those disengaged from care, remains living in the jurisdiction and might be amenable to interventions. Enabling the users of the cascade reports to dynamically adjust inclusion in the cascade based on when people were last seen by the health services proved to be an intuitive and pragmatic approach to restricting the patients reflected as disengaged, in spite of its limitations.

Restricting reports to patients with recent service contact is valuable not only to achieve face validity of distribution on the cascade, but also has important service utility. Faced with an often-overwhelming list of patients who are no longer in care, it may be pragmatic to prioritise those who were more recently in care, as they may be more likely to be contactable and open to interventions to re-engage in care (although the evidence on this is mixed [43–45]). Those with no contact with the health system for a substantial amount of time may have died, re-engaged with private care providers, or moved to a different province where the PHDC cannot track them [46,47].

The Early ART status proved to be a valuable subcategory of patients on ART, given the high proportion of patients who are in this category at any point in time, when including patients restarting ART. Calculations of appropriate ART management usually reliant on determination or estimation of virologic suppression in all of those on ART, could consider this group differently, as people not yet eligible for a VL who are otherwise appropriately managed.

Seven years after the implementation of UTT, knowledge of HIV status in the WC is high, but ART coverage remains well below the 95% target, severely undermining the goal of pursuing treatment as prevention in this population. The overwhelming majority of people not on ART are known to the health services and have experienced and interrupted treatment in the past.

On the other hand, looking at just the numbers of people currently on ART masks the significant movement that is happening into and out of care. While almost half of those on ART have never interrupted treatment, a substantial proportion have experienced an interruption but have successfully returned to care.

Among all individuals on long-term ART—regardless of past disengagement—the proportion virologically suppressed is high. This achievement reflects the effectiveness of contemporary ART regimens, the ability of the healthcare system to provide ART and (at least the minimum) clinical and psychosocial care in a consistent fashion, and the capacity of the vast majority of patients to incorporate health-seeking behaviour and adherence to ART into their daily lives. The on-going successful roll-out of dolutegravir-containing regimens, with associated high levels of viral suppression, is an important achievement. The high proportion of those returning to care with successful viral suppression implies that this is indeed for many a normal cycle of adherent re-engagement that could potentially be facilitated by appropriate programming.

The changes in 2020 and 2021 on the temporal reporting tools demonstrate how the COVID-19 pandemic and government response impacted the HIV programme and also the extent to which the health services were subsequently able to recover. World events that can impact health programmes are not limited to pandemics, and temporal tools like these will assist health services in future to assess the impact of anticipated challenges including local social unrest and severe climate events, and monitor progress to mitigate or recover from their negative impacts.

In this population, disengagement occurred proportionally more in the Early ART period after starting or restarting ART, but absolutely more in the Long-term ART period. As a result, an intervention to reduce disengagement that was targeted at the Early ART period would target individuals at a time of relatively higher risk but miss the majority of those at high risk of disengagement. Substantial disengagement occurred at all points on the cascade, and while not explored statistically in this analysis, there were no obvious stand-out differences in patient characteristics when comparing patients in different statuses. This suggests that an outsize impact of an intervention for patient retention or re-engagement which is targeted based on routinely available patient characteristics, is unlikely. In the context of a mature and generalised HIV epidemic, the focus may need to be on making interventions massively scalable and inclusive, rather than targeted [48].

Notwithstanding these initial observations, as the populated cascade is based on individuated data, it will be possible in future analyses to explore associations with disengagement and re-engagement, to better inform interventions. In addition to routine demographics and clinical data (laboratory results, comorbidities, pregnancy), forthcoming analyses could include comparisons of different geographic locations of home and primary facilities, enrolment in repeat prescription collection strategies, facility staffing ratios, importance of past disengagement, and duration of those treatment interruptions to future disengagement.

This exercise has demonstrated that the objectives of CBS can be met through a service-led consolidated health data environment for person-level health data. The data are immediately available to support direct service delivery, through consolidated shared health records for clinicians, and line lists of patients who require interventions, or management reports tailored to the specific requirements of each level of the health services. It is no longer necessary to advocate for parallel CBS infrastructure which externalises data to third parties, whether in government or outside of government. The historical sentinel events proposed for HIV CBS that enumerate patient identification, linkage to care, ART initiation and virologic testing, are likely to be supplemented or supplanted by the need to track patient care engagement and medicine dispensing longitudinally in an integrated way across all health care services. Just as these results have shown that people engage and disengage with HIV care in a cyclical fashion, it should be anticipated that they will interact with other health care services similarly. The health data system should be organised in a manner that allows for comprehensive monitoring of such engagement and disengagement patterns for all health conditions, alongside supporting integrated shared health records to support clinical care.

The current analysis has demonstrated the value of triangulating data from multiple sources to overcome the absence of definitive single sources or gaps in coverage of individual data sources. On the flip side, there is a critical threshold for coverage of data points by the consolidated data for the approach we have taken to be feasible.

A number of data limitations were highlighted with respect to the definitions of the cyclical cascade statuses, including incomplete evidence of HIV testing and linkage to HIV-specific services. Incomplete evidence of treatment, and imperfect linkage of evidence from different sources for the same individuals, may have led to an overestimate of interruptions.

Among people no longer in care, it is unknown how many have migrated out of the province. The current inability of the health service to access vital registration data was highlighted, as out-of-facility deaths and those in private sector institutions were likely to have been missed [49].

The temporal analysis of this cohort leveraged a proposed cyclical cascade concept to provide insights that may be complementary to those provided by other representations of the cascade and by traditional longitudinal cohort analyses conducted by duration in care or on ART. In so doing, there are questions related to outcomes by duration on ART and temporal trends in outcomes by duration on ART, which were beyond the scope of the current analysis. Duration on ART dimensions will be valuable additions to cyclical cascade reporting.

Notwithstanding a largely homogenous and nationally standardised approach to HIV care in the South African public sector, management of public healthcare is devolved to provincial and in some cases metropolitan governments. As such, these findings in terms of feasibility of the cascade and the characteristics of patients on the cascade may not be fully generalisable to other high HIV-burden settings in South Africa and beyond. It should be noted, however, that the information systems that form the basis of the included analysis are typical of those in the rest of the country, and from an HIV programme performance perspective, the WC is not an outlier, with service characteristics and HIV coverage metrics similar to those in the rest of the country [23].

In conclusion, using routine data it was possible to populate a cyclical cascade of HIV care that captures the nonlinear care journeys of individuals living with HIV. In this generalised mature HIV epidemic, most people are treatment experienced. Disengagement is common and occurs at various points along the cascade, making it challenging to identify high-impact intervention opportunities. While historical HIV cascades remain valuable for target-setting and service monitoring, they can be complemented with insights from more detailed cyclical cascades.

## Supporting information

S1 Appendix Supplementary Technical Appendix. (DOCX)

S1 STROBE Checklist STROBE Statement—Checklist of items that should be included in reports of cohort studies. (DOCX)

## Abbreviations

ART: antiretroviral therapy
CBS: case-based surveillance
COVID-19: Coronavirus Disease 2019
INSTI: integrase strand transfer inhibitor
NNRTI: non-nucleoside analogue reverse transcriptase inhibitor
PHDC: Provincial Health Data Centre
UID: unique health identifier
UTT: universal test and treat
VL: viral load
WC: Western Cape
