# Supplementary material for: The cyclical cascade of HIV care: Temporal care engagement trends within a population-wide cohort
Source: PLoS Med. 2024 May 10;21(5):e1004407. doi: 10.1371/journal.pmed.1004407 (PMC11125544; doi:10.1371/journal.pmed.1004407)
Supplement: S1 Appendix — (DOCX) [file pmed.1004407.s001.docx]

The cyclical cascade of HIV care

Supplementary Technical Appendix
Generating the HIV Cyclical Cascade

24 October 2023

Based on the experience of the Department of Health and Wellness in the Provincial Government of the Western Cape, South Africa, this technical appendix details the available routine data sources which can potentially contribute to understanding which patients have HIV, their care engagement, disengagement and re-engagement patterns, and current treatment status. The paper further details the processes which have been developed to infer and assign a level of confidence in the inference of which patients have HIV (phenotype algorithms), as well as the logic underpinning the assignment of care engagement and treatment outcome status. The final cascade is a key data resource directly supporting individual patient care, service management and operational and epidemiological analyses, meeting all the goals of case-based surveillance.

# Glossary

| HIV | Human Immunodeficiency Virus |
| --- | --- |
| ART | Antiretroviral Therapy |
| HIE | Health Information Exchange |
| PMI | Patient Master Index. This is a master patient repository containing the Provincial unique health identifier as well as patient identifiers. |
| UTT | Universal test and treat |
| RMR | Routine Monthly Reporting |
| Cascade | A cascade provides a comprehensive view of the patient’s health episode including demographics, facilities, key dates, lists of evidence, outcomes and co-morbidities that are relevant for patient management. The cascades are created at a patient level and can act as a patient register for a particular condition. Cascades are optimised for operational and management purposes and are easily aggregated by service area.  Cascades are available at the individual folder level, as well as de-duplicated against the “dominant” folder number. The dominant folder number is assigned using patient matching algorithms. |
| Data Centre / PHDC | The Data Centre or PHDC (Provincial Health Data Centre) is a team of dedicated data scientists, engineers, public health medicine specialists, programmers, administrators and support staff. The PHDC consolidates patient level data from routine health information systems used throughout the WC, facilitating data integration as well as pervasive reporting. The PHDC also develops reports and clinical tools in close collaboration with users on the platform. |
| CBS | Community Based Services |
| WC | Western Cape |

# Background

Within South Africa, public health services are managed at a provincial level, and patients can access primary healthcare services at any public-sector facility free of charge, premised on initial primary care access with appropriate referral to hospital services, except in the case of emergencies. The Western Cape (WC) is one of nine provinces, and due to sustained investment in and stewardship of information systems and infrastructure over the past 25 years, has established some of the core requirements for being able to utilise person-level health data from administrative and routine systems to support direct patient care. The service models and most of the information systems are not however different from those in use in the rest of South Africa, and in many similar settings, and lessons learned in consolidating routine person-level health data in support of HIV care in the WC, could be more widely applicable.

Case-based surveillance is often promoted as a means to utilise person-level health data to overcome the shortcomings of aggregate data systems in poorer countries or data silos in better resourced settings,[1, 2] frequently requiring extraction of key case events for consolidation in a dedicated case-based surveillance system. The relatively uniform nature of public sector service provision in South Africa under a single authority, also means that the consolidation can happen as part of the service remit of the government health services, meeting all the requirements of case-based surveillance while simultaneously functioning as a Health Information Exchange, directly supporting individual patient care, without the need for a separate dedicated surveillance system.

This working paper seeks to describe all aspects of assembling what is termed the person-level “HIV cascade”, a virtual cohort enumerated based on a form of phenotype algorithm, and which is the basis of “pervasive reporting” through which this single consolidated cohort can support a range of patient, clinician, manager and researcher needs.

## Background to HIV services in the WC

Public sector antiretroviral therapy (ART) provision started in the WC in 2001, with the Khayelitsha HIV services providing ART through a partnership between Provincial and City health services and Médcins Sans Frontières.[3] In 2004 ART, was formally launched as a national public sector intervention, with increasing availability over time reflected in both evolving eligibility guidelines and increasing uptake, culminating in universal ART for all patients with HIV.

**HIV testing**

While some patients are diagnosed in hospitals or through formal laboratory testing, the mainstay of HIV testing is through point-of-care rapid testing, with positive tests being confirmed by a different rapid test, and equivocal or discordant rapid tests in the same patient being confirmed through laboratory testing. HIV rapid test results are not always digitised (recorded in the paper-based medical record), and this was especially the case in earlier years.

**Clinical treatment services**

Until April 2010 only patients with a CD4 CD4+ T-lymphocyte cell count (CD4) less than 200 cells/µl or a stage IV illness (excluding extra-pulmonary tuberculosis [TB]) were eligible for ART. Between April 2010 and August 2011 pregnant women and patients with TB were additionally eligible for ART if they had a CD4 count between 200 and 350 cells/µl, at which point all patients with a CD4 count below 350 cells/µl became eligible for ART. In January 2015 the CD4 eligibility threshold was increased to 500 cells/µl, prior to Universal Test and Treat (UTT) in September 2016. There was a similar evolution in recommended antiretrovirals for first, second and later third line treatment, in the prescribed routine effectiveness and safety monitoring, and in the evolution of service models to optimally manage an ever-increasing number of patients in life-long care, with differing needs for support. These have been extensively described in various publications. [4, 5]

Currently,[6] the first line regimen is a fixed-dose once-daily combination of tenofovir, lamivudine and dolutegravir, with zidovudine replacing tenofovir and a protease inhibitor replacing dolutegravir in second-line treatment. Third-line treatment is based on resistance testing and the recommendations of a clinical committee. Virological monitoring is annual with an additional early test in the first 6 months of treatment to assess initial response. Patients with elevated viral loads (above 1000 copies per ml) receive adherence interventions and more frequent virologic monitoring, and are considered encounteto have failed a regimen if viraemic on consecutive tests despite adherence interventions.

Whereas CD4 counts were initially annually indicated in patients pre-ART and six-monthly once on ART, patients who are durably virologically suppressed (for at least a year) are no longer routinely monitored through CD4 count testing. Baseline CD4 counts are still indicated in all newly diagnosed patients, and the first CD4 count is often the first digital evidence of HIV that is available.

**Service delivery models**

Over time ART access has been expanded in part through an expansion of where ART could be accessed, from selected primary care facilities and hospitals initially, to almost universal access in primary care sites. Adherence clubs were pioneered in part in the WC as an early differentiated model of care (DMOC),[7, 8] enabled by the Chronic Dispensing Unit (CDU), a longstanding high throughput pre-packaging facility which prepares and parcels medicines for named patients (usually for two months at a time) and delivers them to the dispensing site, often boxed by club to improve efficiency. This has become an important dispensing route in the WC, accounting for a high proportion of medicines dispensed, especially for stable long-term patients. Most CDU parcel collections and clubs are still predominantly facility-based, but there have been many smaller innovations, including dedicated clubs for pregnant women, adolescents and other groups, quick pick-up dispensing options, electronic lockers, last mile delivery through social enterprises (including by bicycle), amongst others.

**Prevention services**

Predating ART as treatment the WC initiated the use of ART to prevent vertical transmission of HIV from mothers to their children,[9] again through evolving protocols until universal access to lifelong ART. Post-exposure prophylaxis has long been available, though is not formally tracked on a per-patient basis. Pre-exposure prophylaxis is now available but has not been implemented or taken up as widely as had been hoped given the ongoing high incidence. Voluntary male medical circumcision has been a further priority prevention intervention, although this too is not routinely tracked digitally on a named-patient basis.

**Co-morbidities**

Patients with advanced HIV disease (eg. Adults with a CD4 count < 200 cells/µl) receive cotrimoxazole prophylaxis, and those who have had cryptococcal disease receive fluconazole prophylaxis. All HIV-infected patients are eligible for TB preventive therapy, historically as 12 months of isoniazid, although new shorter regimens are being introduced. There is a high prevalence of multi-morbidity in patients with HIV, and many patients with HIV are also receiving care for other high burden conditions such as hypertension and diabetes, in addition to the high incidence of TB in patients with HIV.

## Background to HIV treatment monitoring

Initially HIV treatment was monitored through paper-based registers, with the exception of a number of partner-supported projects which maintained electronic registers. This included the EKAPA database in the early Khayelitsha program, which was later redeveloped as part of the provincial government-managed Primary Health Care Information System (PHCIS – separate EKAPA module launched in 2008, and later fully merged into PHCIS). Paper-based registers were digitised around 2010, and due to connectivity challenges at the time, many of them were digitised into an off-line electronic register (TIER.Net) which later because the national HIV and TB electronic register, and is still utilised for HIV program monitoring in some sites in the WC.[10]

The hospital information system in the WC, Clinicom, was gradually implemented between 1998 and 2017, finally reaching every hospital in the WC and sharing a single patient master index and unique patient identifier (known colloquially in the WC as the Clinicom number). From 2007 onwards primary health care systems in the WC and the city started linking to the same number, through interoperability with Clinicom. Whereas Clinicom always included ICD10 coding, the reliability and completeness has always been suboptimal due to there being no claims reliant on accurate coding. The introduction of an electronic discharge summary in 2016 improved coding completeness and accuracy, but not universally. In addition to the CDU described above, acute dispensing of medicines, including antiretrovirals, in hospitals and latterly in large PHC clinics, has been digitised through an electronic dispensing application (JAC).

The availability of the unique identifier on printed bar-coded labels affixed to all clinical stationery and request forms has ensured that the majority of laboratory results, pharmacy dispensing records and TIER.Net register entries are linkable back to a folder number, contributing to the ability to link data across systems. It is not uncommon for patients to have more than one identifier in spite of workflows designed to prevent this, but due to robust civil birth registration, dates of birth and often civil identifiers are very useful additional identifiers for linkage.

Whereas most routine reporting has been based on aggregate monthly or quarterly outputs from one of the register-based systems, such as TIER.Net, PHCIS or the City of Cape Town Patient Record Health Management Information System (PReHMIS), there have been persistent and growing concerns that these systems do not adequately account for patient movement between facilities, and are often missing key outcome information (laboratory results and information on patients who have died). The same challenges are faced with facility-level line list reports intended to assist with patient management, such as those meant to identify patients newly lost to follow-up, missing viral loads, or viraemic. One of the key objectives of the HIV cascade described in this paper is to provide better data to assist in direct patient management (identification of the patients who would benefit most from proactive intervention), and improved management data on program performance (more complete and accurate uptake and outcome data).

## Background to the Provincial Health Data Centre

As described above, to ensure continuity of care across facilities and to enable ease of record linkage, a unique health identifier was established in the WC.[11] The WC has sustained investments in health information systems for decades, including patient registration systems, the unique health identifier and the development and maturation of routinely used digital health systems. When a patient presents at a health facility, a unique patient identifier (folder number) is either retrieved or generated for them depending on whether they have or have not accessed services before. As the patient accesses various services within the health facilities, their folder number uniquely identifies the patient within each of the routine health information systems that support those services. The WC Provincial Health Data Centre (PHDC) is a health information exchange (HIE) that integrates person-level administrative and clinical data from these routine health information systems into one consolidated environment that leverages the unique health identifier for linking person-level data across systems. The PHDC exposes the data as actionable outputs including patient level alerts, a clinical viewing application called Single Patient Viewer (SPV), patient-level line listing reports, and aggregated management reports and dashboards. The structure and history of the PHDC has been described in detail.[12]


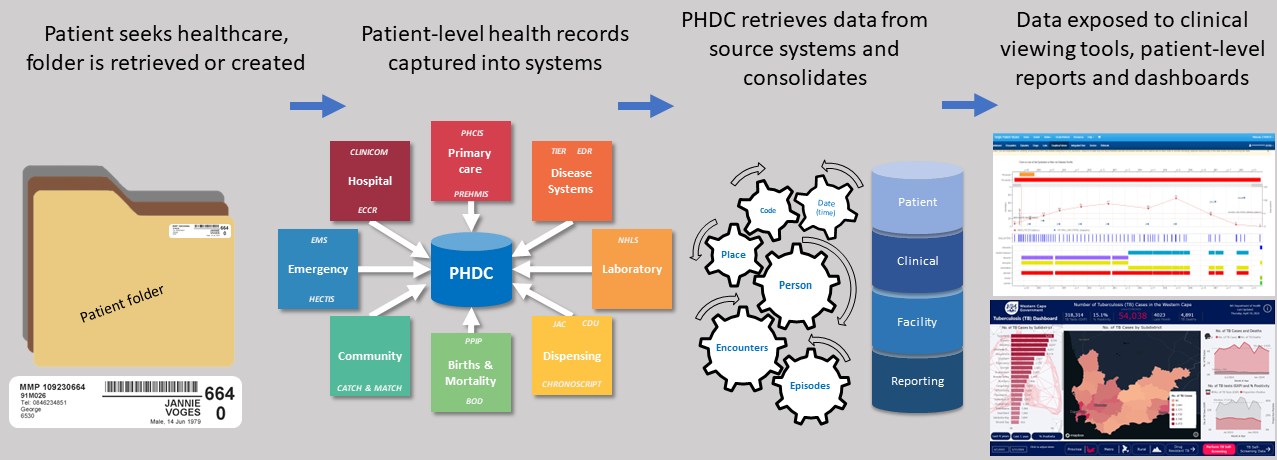
Fig A in S1 Appendix. Illustration of data transfer and integration within the PHDC

## Rationale for pervasive reporting

The utilisation of integrated data from multiple sources across multiple facilities is self-evidently valuable for patient management. It is only possible due to the coverage and success of existing routine health information systems. The alternatives are one monolithic system, or multiple systems each having to replicate bidirectional interoperability and reporting. The WC has selected the health exchange and interoperability approach with pervasive reporting over the alternatives, in line with the Health Normative Standards Framework (HNSF) and best practice architectures globally.[13] However, integrating data from multiple sources is more complex than using a single source, and one must explicitly accept that there may be multiple conflicting data points for the same event, versus the simplicity of utilising one data source, even if frequently inaccurate. Figure 2 illustrates the long-standing vision of supported self-management, in which patients would be able to access services through a variety of service delivery channels, but the backend data systems would be able to identify who was in need of intervention, alleviating the need for everyone to regularly attend a specified fixed health service. Core to the model is the digital safety net, and it was always envisioned that this would be underpinned by a call centre. The current plans for call centre functionality in the WC now underscore the readiness for this model.


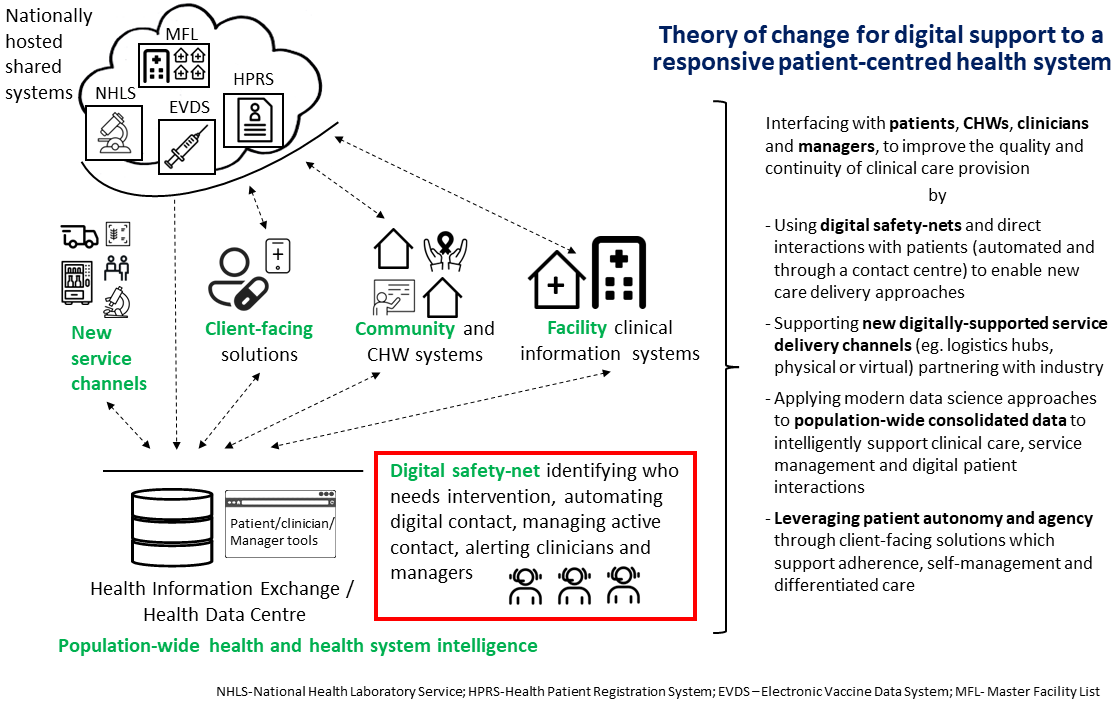
Fig B in S1 Appendix. Theory of change for digital support to a responsive patient-centred health system

At present, each health facility offering HIV services has one primary system for HIV patient management and data collection (they either use one of the online systems PHCIS or PREHMIS or the offline system TIER.Net).[10] These systems provide individuated reports that can be used for individual patient management. Aggregated reporting is done uniformly, whereby each system extracts data into TIER.Net compatible dispatches which get combined at a subdistrict, district and finally provincial level, from which aggregated reports can be run that are comparable with other provinces.

Pervasive reporting will have a meaningful impact on data capture processes, aggregate statistics and HIV retention in care. Even though individual facilities send all lab samples to the National Health Laboratory Service (NHLS), and many of the facilities use electronic dispensing systems, such as JAC, or have patients enrolled with the Chronic Dispensing Unit (CDU), the clerks still capture the laboratory and drug dispensing data into the HIV patient management system in use at their facility. Data capturing is an error-prone and time-consuming task that could be alleviated, especially for laboratory data which are more complete than the captured laboratory data.

In addition to alleviating unnecessary data capture, pervasive reporting can assist with refining lists of patients that are currently actively seeking health services in the facility (action lists). Silent transfers can be identified and removed from any facility’s list of patients requiring follow-up, which often accounts for differences between a source-system and a pervasive list. Similarly, soft outcomes such as Transfer Out (TFO) or Lost to Follow-up (LTF) can be verified or refuted by data from other facilities, or preferably dynamically ascertained from the raw data. Hard outcomes (such as death) can be derived from other sources (hospital admissions, death registry) that are linked within the HIE. Finally, facility-allocation can be based on more passive data sources, rather than relying only on active capture on a register.

By design, pervasive reports and source-system single-facility reports will differ, but by ensuring that (1) data acquisition from the source systems is accurate and timely, (2) data are pre-processed and conflicting data points prioritised appropriately, and (3) indicator and data element algorithm logic is well defined and transparent, we should be able to rapidly identify the source and reasons for patient statuses within reports. In the sections that follow, the processes in place for source data integration, episode inference, report data pre-processing, and indicator logic pertaining to HIV outputs will be described.

# Data harmonisation

## Introduction to data harmonisation within the PHDC

All sources follow a similar pattern of data import and integration, which we call “ETL” (Extract, Transform and Load). This pattern is illustrated in Figure 3. First, we extract the data from a source system. We temporarily store a copy of the raw extracted data in our Staging database and permanently store a copy of the raw data in an archiving database that can easily be referred to. The copy in our Staging database undergoes data cleaning, mapping and transformations. This includes patient matching, facility matching, and mapping to a common coding system where possible, which are described in more detail in the sections that follow. After the data has been transformed, the data are loaded into standardised structures in our Clinical and Patient databases. Thereafter the PHDC infers episodes (health conditions) that a patient has, as well as encounters, which are visits inferred from electronically captured visits as well as data such as lab results and drug dispensing that can be used to show that a patient was at a facility, even if their visit was not captured. Finally various indicators are calculated from the data in the Clinical database and consolidated into patient-level cascade tables stored in our Reporting database, which are the structures underlying the outputs.


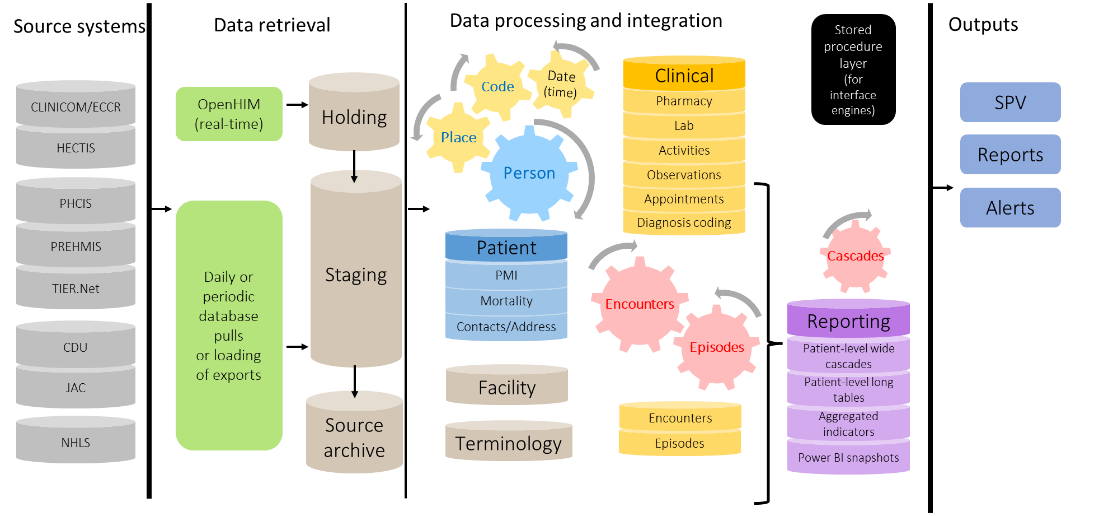


Fig C in S1 Appendix. Integration of source data into the PHDC data structures

## Shared components of source data integration

### Facility curation

Most of the systems use their own facility codes and facility names. We carefully map these facilities to the master facility list in SINJANI (DHIS equivalent). If any new facility descriptions or codes from the source system are identified during a data load, they are included in a daily report that prompts us to curate the mapping. We store a link to the source facility code and name against each record so that if we need to remap the facility, we can easily do so.

### Patient Matching

The WC has a Patient Master Index (PMI), which is the master directory of all patient identifiers that stores and maintains the unique health identifier known as the “folder number”. Hospital and primary care patient registration systems connect to the central PMI and are able to send updates or add new patients. The PHDC stores a copy of the PMI locally, and stores the identifiers received for each patient from every source system, as sometimes the details associated with the same folder number differ slightly between sources. When data is received from a data source, a patient matching algorithm is run on the data in order to match the individual records to the PMI and assign the appropriate folder number. The patient matching algorithm requires that at least the folder number and one other identifier match the record in our PMI (e.g., folder number and date of birth), or that the national identifier number matches, or that a combination of names, date of birth and physical address match.


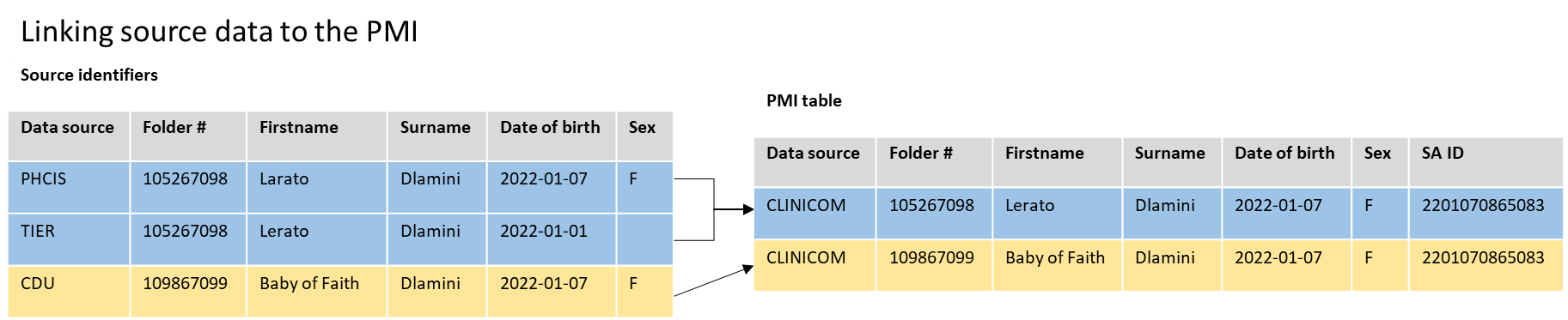
Fig D in S1 Appendix. Linking source data to the Patient Master Index

In addition to matching source data to the PMI, patient matching algorithms are run on the PMI itself to link folder numbers with sufficiently similar other patient details and thereby “de-duplicate” the PMI. For example, two folder numbers with the same national identifier would be linked to each other. As part of this de-duplication, we assign one of the folder numbers in the linked set as the “dominant” folder number. This linkage is a dynamic mapping, data captured against individual folder numbers are kept separately and only merged in visualisations if explicitly requested in a report or in the single patient viewer. For ease of data retrieval, we pre-merge the episode and cascade data so that all relevant data across any linked folders are combined into one record against the dominant folder.


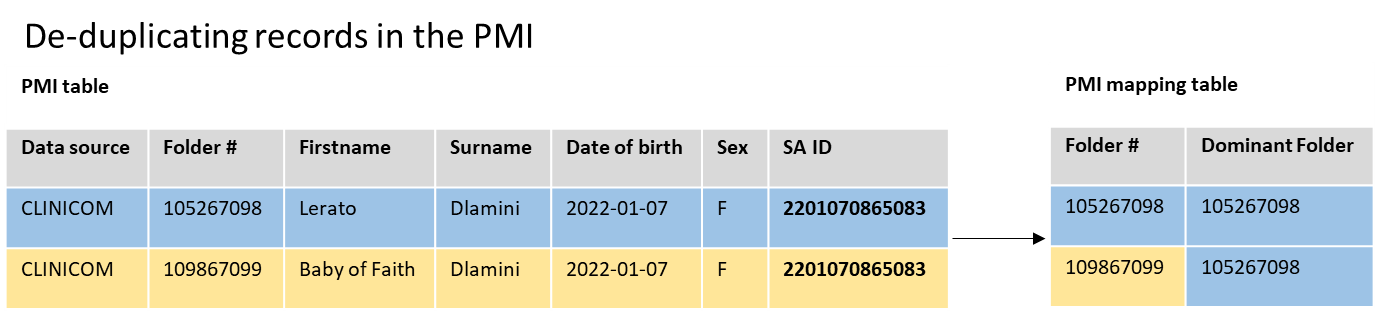


Fig E in S1 Appendix. De-duplicating records within the Patient Master Index

The patient matching algorithm is currently deterministic and based on a hierarchy, such that the strongest rules are assessed first, and the first match is accepted. Additional work is being done to investigate the benefit of applying machine learning algorithms and graph theory to linkage.

## Core data sources

The PHDC integrates data from over 40 sources of clinical and patient data from routine systems used in the WC, of which over 20 sources are integrated daily. These data sources include data collected or generated by laboratory tests, drug dispensing, primary care visits, hospital admissions and outpatient visits, disease monitoring systems, and community visits, which have been described in detail elsewhere.[12] The core routine data sources that feed into the HIV episode and cascade are described below.

### Pharmacy data sources

Drug dispensing information is collated from two pharmacy sources JAC and CDU (Chronic Dispensary Unit). We also include drug data from primary care and disease information sources which are described later in this document. Under each source the following will be covered: Linkage, Coverage, Frequency of integration, Inference of encounters.

#### JAC

**JAC** is a dispensing system that is used in hospitals and several primary care sites, for which we have data from 2010. **Linkage:** JAC has online access to the Provincial PMI, and as such most records can be linked to a unique identifier. For the 2021 calendar year 99.99% of records linked to the PMI. **Coverage:** JAC data has been received from 122 facilities in total, and 113 distinct facilities since January 2021. This includes 64 facilities in the Cape Metro (28%), 12 in the Cape Winelands (11%), 13 in the Overberg (21%), 13 in the Garden Route (11%), 8 in the West Coast (8%) and 3 in the Central Karoo districts (9%) respectively. **Frequency of integration:** We integrate JAC data daily, and there is usually at most a 2-day lag in data integration. **Drug returns:** We integrate all JAC dispensing records that were dispensed to an individual, as well as JAC returns. The JAC returns are logged as a separate line of information in the source. To easily identify issues that were returned, we update the original dispensing record with a flag that indicates the medication was returned. **Drug durations:** We do not receive directions for use or duration information from JAC. We have logged a development task with the provider that manages JAC to include the duration in the data source that we have access to. We currently estimate the duration of dispensing based on the number of packets or pills that were dispensed. If the pack contains a multiple of 30 pills or 28 pills, the duration is set to 30 days or 28 days, respectively. If there is no pack information, and the quantity of pills is a multiple of 30 or 28, the duration is also set to 30 or 28 days respectively, alternatively the duration is set to the quantity. This will be updated as soon as we start to receive dosage instructions. **Inference of encounters:** We infer primary care encounters from all JAC dispensing events where the drugs were not returned. We only create primary care encounters if one does not already get recorded into a primary care information system. We do not infer hospital encounters from JAC as patients should be captured into the hospital information system, CLINICOM, and may have many drug dispensing records during their hospital visit that would unnecessarily balloon the encounters records. **Drug code mapping**: We receive the drug name, strength, and a code that corresponds with the WHO Anatomical, Therapeutic and Chemical (ATC) drug classification system. These drug names and codes are carefully checked and mapped to the latest ATC reference table stored within the PHDC. Over the years, 103 unique drug names and 33 unique codes have been received for HIV antiretrovirals, which have been mapped to 41 unique ATC codes.

#### CDU

**CDU** is a chronic medicine dispensing system used across the WC for patients who are eligible to receive their chronic medication pre-packed, for which we have data from 2012. ART regimens are typically mapped to a fixed dose combination unless individual drugs have been scripted. **Collections and verifications:** CDU provide us with information about drugs that were packaged for collection and the anticipated collection date, how many repeats the patient would be collecting. From 2020 we additionally started receiving notification that the parcels have been collected via “manifest returns”. We flag any script that has been collected with a “verified” flag. **Linkage:** CDU does not connect directly to the PMI, which slightly impacts linkage, however most records do link to the PMI. For the 2021 calendar year, 99.51% of records linked to the PMI. **Coverage:** CDU data has been received from 306 facilities in total, and 264 unique facilities since January 2021. This includes 101 facilities in the Cape Metro (44%), 53 in the Garden Route (44%), 40 in the Cape Winelands (35%), 34 in the West Coast (35%), 16 in the Overberg (25%), and 13 in the Central Karoo (37%) districts respectively. **Frequency of integration:** Collections are integrated at least weekly, although we have started receiving data more frequently and we are working to import it as soon as it is received. Manifest returns are integrated every 1 or 2 weeks depending on when we receive the data. **Drug durations:** From CDU we receive one line per repeat provided, which we condense into one line of data with the total number of repeats provided. For CDU, we multiply the number of repeats by 28 days to get the duration the patients were dispensed medication for. **Inference of encounters:** We infer primary care encounters from all CDU manifest return records. This is necessary as drug collections stopped being digitised as head counts during early 2020, in a move to reduce burden of data capture and improve ascertainment of drug collections The encounters created from manifest returns are aggregated monthly from the PHDC and imported into SINJANI for aggregated reporting. **Drug code mapping**: We receive the drug name, and a category code that is in the format of the British National Formulary system (BNF). These drug names and codes are carefully checked and mapped to the latest ATC reference table stored within the PHDC. Over the years, 124 unique drug name and code combinations and 85 unique codes have been received for HIV antiretrovirals, which have been mapped to 19 unique ATC codes.

### Primary care and disease information systems

In addition to the pharmacy sources, we receive drug information from the HIV registers within the Primary Care information systems (PHCIS and PREHMIS used by sites managed by the Provincial Health Department and the City of Cape Town Metropolitan Municipality respectively) as well as the disease information system, TIER.Net.

#### TIER.Net

**TIER.Net** (Three interlinked Electronic Registers) is an offline, facility-based system that is used to capture HIV data. Historically, all sites used TIER.Net, to capture HIV data. These data were then combined quarterly at a subdistrict, district, and provincial level for national reporting. As the primary care sites moved toward using online systems for patient management, capability to capture HIV information was built in and instead data are sent from the online systems to the provincial TIER.Net quarterly. **Frequency of integration:** We receive TIER.Net data weekly for a set of sites in the City of Cape Town Metropolitan municipality that do not use PHCIS or PREHMIS yet. For the other sites we receive data via the Provincial dataset that is updated quarterly. At present, each TIER.Net load (weekly or quarterly) involves a complete delete and reload of all the existing TIER.Net data for the included facilities. We are working on changing this to be an update of changes to the existing data and insert of new data to reduce the processing times. There is also an ongoing project to set up bidirectional transfer between sites using TIER.Net and the PHDC using the LHIM (Local-Health Information Mediator. **Data types that are integrated:** TIER.Net data are integrated into our pharmacy, observations, encounters data structures. We do not integrate lab data from TIER.Net as we receive this from NHLS. We are currently working on the import of appointment data. **Drug data integration:** All ART records are incorporated into the pharmacy table if the exact record is not present in one of the other systems. We receive the data from TIER.Net as a single line with up to 5 columns containing individual drugs that are part of the patients’ treatment regimen. We split that line up into one line per individual drug dispensed, which we map to the relevant ATC code. We exclude all records that were imputed by TIER.Net and exclude records where the Regimen Code is listed as “Did not attend” or “Stopped’. In addition, only records where the regimen date is after the ART start date recorded and not recorded in the future are included. We intend to only import TIER.Net data for specific facilities that do not have PHCIS or PREHMIS as sometimes there are duplicates in the TIER.Net database caused by issues in the quarterly updates from the online systems. **Drug durations:** The drug durations in TIER are provided as months dispensed, we multiply these months by 28 days to estimate the days the medication was dispensed for. **Linkage:** TIER is an off-line system, which impacts linkage, however most records do link to the PMI. Based on the data in the pharmacy table, for the 2021 calendar year, 98.49% of pharmacy records from TIER sites linked to the PMI. **Inference of encounters:** We infer primary care encounters from all visits recorded into TIER.Net, where the same encounter has not been reported by any of the other primary care systems. Hospital encounters are not inferred, as we may artificially balloon the data already captured into Clinicom. **Observations:** All form entry data from TIER.Net (e.g., WHO stage at ART start, facility-level outcomes such as transfers, etc.) are stored in an observations data structure. Even if the observation is present in another system, the TIER.Net observations are retained.

#### PHCIS

**PHCIS** (Primary Healthcare Information System) is the online primary care information system used within sites managed by the ProvincialDepartment of Health. PHCIS data are arranged into separate tables per domain, similar to the PHDC Clinical database (i.e. separate tables for visits, medication, episodes etc., refer to Figure 6). Most of the data in PHCIS are captured through workflows that link the data to specific episodes, enabling us to identify what condition the data were captured against. All updates to PHCIS are identifiable via newer modified dates and deletes are identifiable through active flags on the PHCIS tables. We retain all versions of the data we receive from PHCIS, with the most recent version flagged in our Archive database. **Frequency of integration**: Every day we receive the data that were captured into PHCIS on the previous day. Data are pulled from the PHCIS databases into the PHDC databases by running an SSIS package which is executed as part of the PHDC daily load procedure. Any delays to integration are typically as a result of delays in capturing. **Data types that are integrated:** PHCIS data are integrated into our pharmacy, observations, encounters, activities and appointments data structures. We do not integrate laboratory data from PHCIS. We send the NHLS data to PHCIS for integration into the source system, though this is still under development. **Drug data integration:** All drug records are incorporated into the pharmacy table along with the episode they were dispensed for. PHCIS data typically includes one line per drug dispensed. We restrict imports to medication recorded with the following statuses: “Begin”,”Continue”,”Restart”,”Occasional issue”, and ”PMTCT single drug”. At present, if the same dispensing record is available in JAC, the JAC record is updated to include the PHCIS information, although in future we intend to allow duplication between sources in the primary pharmacy table to enable rigorous comparison to the source systems. **Drug durations:** In PHCIS, the number of days dispensed is recorded which is used as the duration. **Linkage:** Because PHCIS is an online system that links to the PMI, all records should link to the PMI, however we do perform some minor checks on the data and a fraction of patients may be unlinked. Using the pharmacy data, For the 2021 calendar year, 99.996% of pharmacy records from PHCIS sites linked to the PMI. **Inference of encounters:** We infer primary care encounters from all visits recorded into PHCIS either via the attendance records or via the RMR data capture. PHCIS and PREHMIS have the highest priority over other inferred primary care encounters as they provide visits captured into a patient registration system. **Observations:** All form entry data from PHCIS (e.g., WHO stage at ART start, facility-level outcomes such as transfers, etc.) are stored in an observations data structure. The observation date is typically set to the encounter date. **Appointments:** All attendance records where the date created is before the attendance date are classified as appointments in PHCIS. Only appointments up to three years in the future are imported. The appointments from PHCIS are stored in our appointments data structure, which specifies the episode for which the appointment was created. **Activities:** We store any RMR (routine monthly reporting) indicator data in the “activities” data structure. These data are typically bulk scanned, although in some cases they are captured from an individual folder. The indicators are determined in line with National and Provincial reporting requirements.


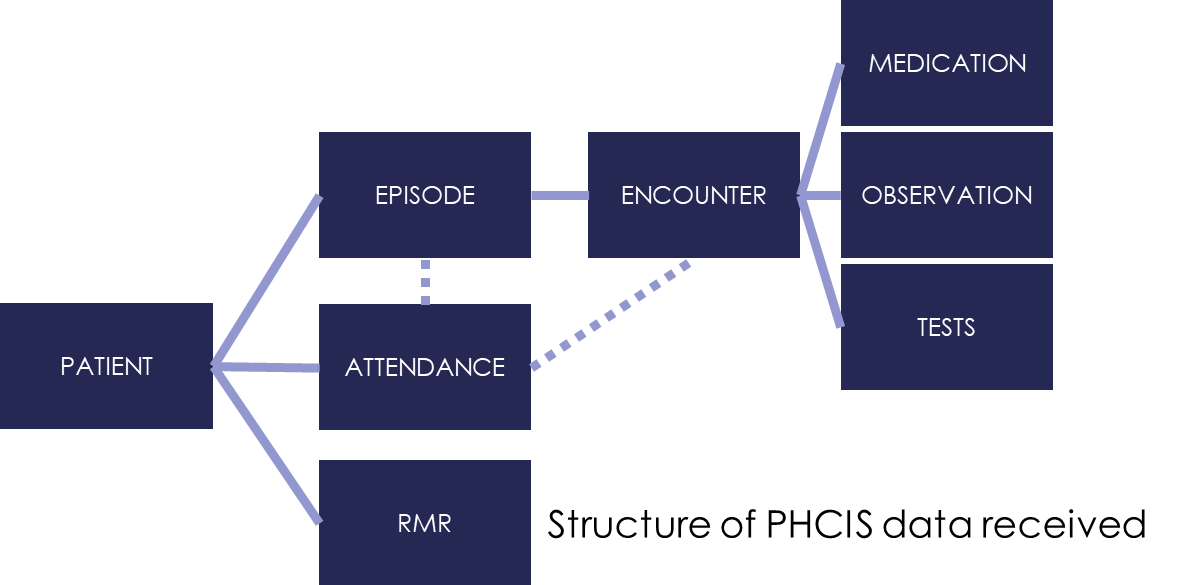


Fig F in S1 Appendix. Structure of the PHCIS data received by the PHDC

#### PREHMIS

**PREHMIS** (Patient Record and Health Management Information System), is the online primary care information system used within sites managed by the City of Cape Town Metropolitan Municipality. The data from PREHMIS are received in an element attribute value (EAV) format, with one line per data element captured per event. The element definitions enable us to ascertain which episode type they are likely to be related to. Updates are processed as deleted and re-entry within PREHMIS. We keep all versions of the data we receive in our Archive database, with the most recent record flagged. **Frequency of integration**: Every day we receive the data that were captured into PREHMIS on the previous day. We receive the data as a flat file that is placed on the PHDC FTP site. If the file is placed on our FTP server after our daily run we will only pick it up the following day, as such there may be up to 2 days delay prior to integration. **Data types that are integrated:** PREHMIS data are integrated into our pharmacy, observations, encounters, and activities data structures. We only receive PREHMIS data for Headcounts, HIV, TB and a few antenatal elements under the current data sharing agreement, which is currently being revised. **Drug data integration:** All drug records are incorporated into the pharmacy table. PREHMIS does not include the actual drug dispensed at each visit, but rather the fact that a regimen was provided. The first regimen is provided and any subsequent changes to the regimen are provided. We then fill in the exact regimen by looking backward to the last change / starting regimen. There is a small subset of patients for which a regimen was never captured into PREHMIS. We try to extract these from the historical TIER.Net data. However, if we are still unable to identify the regimen we simply indicate that an “Unknown ART Regimen” was provided. This affects roughly 0.65% of all dispensing records received from PREHMIS. **Drug durations:** The drug durations in PREHMIS are provided as months dispensed, we multiply these months by 28 days to estimate the days the medication was dispensed for. If a patient receives multiple “regimen provided” elements on the same day, we sum these up to calculate the total months they were dispensed medication for. **Linkage:** Because PREHMIS is an online system that links to the PMI, all records should link to the PMI, however we do perform some minor checks on the data and a fraction of patients may be unlinked. Using the pharmacy data, For the 2021 calendar year, 99.97% of pharmacy records from PREHMIS sites linked to the PMI. **Inference of encounters:** We infer primary care encounters from all visits recorded into PREHMIS as a “headcount”. PHCIS and PREHMIS have the highest priority over other inferred primary care encounters as they provide visits captured into a patient registration system. **Observations:** All form entry data from PREHMIS (e.g., WHO stage at ART start, facility-level outcomes such as transfers, etc.) are stored in an observations data structure. **Activities:** We store any RMR (routine monthly reporting) indicator data in the “activities” data structure. Any PREHMIS elements that have been mapped to the activity types that are listed in SINJANI are included.

### Laboratory

While some laboratory data are captured into the primary care and disease information systems, we rely on the laboratory data we receive from the **National Health Laboratory Services** (NHLS) for our laboratory test information. The data includes HIV tests, Viral load tests and CD4 tests, however point-of-care (POC) testing is not routinely included. If point of care testing is received from one of our source systems, it is stored in our Observations or Activities data structure . **Linkage:** NHLS covers all primary care and hospital sites and has excellent linkage to the PMI as facilities send the lab test requests with the unique identifier attached. Over 99% of records from sites that we should expect to link to the PMI do link to the PMI. **Inference of encounters:** We infer primary care encounters from NHLS where there is no other record of encounter. Over the past 4 years, on average 4% of primary care encounters are inferred from laboratory data.

### Hospital and Emergency Centre information systems

#### CLINICOM, ECCR and HECTIS

**CLINICOM** is a hospital system used in all public hospitals in the WC, which provides admissions, outpatient visits and diagnosis and procedure coding. Linked to CLINICOM is ECCR (the electronic continuity of care record), which provides clinician captured diagnosis codes and discharge summaries. **HECTIS** is used at emergency centres in hospitals and CHCs. **Frequency of integration**: Data that were captured the previous day are integrated. **Linkage:** All data from CLINICOM and ECCR link to the PMI as it is a PMI integrated source. **Data integrated into the HIV episode/cascade:** Diagnosis coding information is utilised at a low confidence for HIV. CLINICOM is our primary source of death data, as we only currently routinely receive deaths that occurred in-hospital.

## Data curation and complexities

The primary challenge presented by integrating different data sources is that data that present the same or similar concept are often captured or shared completely differently between sources. Extensive curation is done prior to loading the data into intermediate Clinical data structures (laboratory, pharmacy, diagnosis codes, encounters, observations etc.) so that the data can be used downstream. Systems have been put in place to detect new data that requires curation.

### Laboratory data

The laboratory data includes thousands of test codes and for each test code, several parameter codes. Within the NHLS, the laboratory information system changed in 2015 from DISA to TRAK, and this shift included a complete change in codes. The PHDC mapped all the DISA codes to their corresponding equivalent TRAK codes and uses these in the laboratory data structure. In addition, where possible the codes are mapped to LOINC. However, there are also multiple tests within TRAK that are equivalent, and the LOINC system is very detailed and does not include an intuitive hierarchy. The PHDC has developed an in-house categorisation for laboratory codes called “common concepts”. The key common concepts for HIV are: HIV Viral Load, HIV Serology Result (further broken down into common methods of Rapid, Elisa and POC Rapid), HIV PCR Result, HIV Test Result, Absolute CD4, HIVDR Result. In addition, results are mapped to common results so that they can be standardised – e.g. “P”, “+”, and “Positive” are all mapped to the common result of “Positive”. Viral load results are further standardised by ensuring they always have an assigned numerical value, as sometimes the result is reported as “Lower than detectable limit” (for these we assign a value of 24), and other times the result is reported as being greater or less than a specific value we assign a value of 1% more or less than that value.

The NHLS data is not received directly from the TRAK system, but rather from two separate feeds that we integrate. The first data feed is from the NHLS Corporate Data Warehouse (CDW) and the second is the Health Systems Trust Operational Data Store (HST ODS). We have identified that a small percentage of results are missing from both data feeds, though by triangulation an even smaller percentage are completely absent from the PHDC. We are working with the data providers to resolve any missingness.

### Pharmacy data

The pharmacy data sources each have their own format of coding drugs, which the PHDC has mapped to the World Health Organisation’s (WHO) Anatomic, Therapeutic and Chemical (ATC) classification system. JAC and CDU typically record the combination of drugs that were dispensed, whereas the other sources record the regimen provided, which is split up into separate drug components. We have a mapping between all the ATC combinations and the individual components so that changes in regimen can be tracked over time. One of the challenges with ART regimens is that the patient’s ART can be recorded in multiple systems (e.g., JAC and PHCIS), but the regimens recorded can differ. To partially mitigate this we are adjusting the imports to limit ART data to one primary care/disease information system per facility per time range. However, differences between CDU or JAC and the primary care/disease information system may still occur and these can be difficult to navigate. Furthermore, the collection date in CDU may differ slightly from the actual visit date in the primary care system when the drug was recorded, which causes duplicates. PREHMIS does not indicate the regimen dispensed at each visit, instead they indicate the starting regimen and any regimen changes, so the drugs dispensed are inferred from the most recently recorded regimen.

Sources also have different methods of recording durations. Slight deviations in duration affect the calculations of the last ART in hand date. Facilities have different mechanisms of capturing durations. It is expected that the facilities will enter the full duration prescribed into the primary care system, however during the festive season we have observed that some facilities captured only the additional medication provided to what was provided in JAC, but we cannot sum the durations as this is not used across the board. Furthermore, we have observed that some medications are captured without durations, and the assumption from the facility is that we should rely on the next appointment date that was captured. We intend to calculate at least a conservative and an optimistic ART in hand date so that users can choose to filter outputs as required.

### Encounters data

Visits are captured at all levels of care. To fill gaps in capture, we supplement these captured visits with visits inferred from other activities (e.g., lab tests and drug dispensing) into a data structure called “encounters”. While every effort is made to prevent duplicates, at times event dates captured deviate from the date the drug was dispensed or lab test performed which results in mismatched dates and duplicates. In addition, we only infer encounters from CDU data where confirmation of collection was received. This means there are potential visits that are missing from encounters where drugs were collected but a visit was not captured. It can also result in false visits where packages were collected by relatives or inaccurately processed as being collected. Encounters typically do not carry condition specific information, as patients may be treated for multiple conditions during the same encounter. We use encounters to infer whether the patient is still active in the public sector.

### Appointments data

Next appointments are routinely captured into PHCIS. We store the appointment date, facility and reason for the appointment. In PHCIS, appointments are captured against an episode, so we can for example extract all HIV related appointments. We also store appointments from CLINICOM.

### Observations and activities

During primary care encounters, certain elements are scanned or captured into the primary care systems that are typically aggregated for routine monthly reporting (RMR) into SINJANI. We call these elements “activities”, as they typically describe an activity at the facility, for example “Rapid HIV Test Performed” or “HIV positive (new) client screened for TB”.

In addition, we receive both clinical observations and “questionnaire-response” type information which we store in a data structure called observations. This structure is in “element-attribute-value" format and stores a wide range of data, including blood pressure, height, weight, ART start date, WHO stage at ART start etc. When a patient has multiple observations, at present the observation is prioritised with the lowest data source ID and earliest observation date. This means that observations recorded into TIER.Net (data source ID = 5) will be prioritised over observations in PHCIS and PREHMIS (data source ID of 12 and 11 respectively). Though conflicting observations will be rare, improvements to this prioritisation logic will be made, such that different observations types can be prioritised individually based on separate rules. For example, the most recent WHO stage would be more beneficial than the earliest regardless of the data source, and PHCIS and PREHMIS should be prioritised over TIER.Net if there are multiple observations on the same day.

# The HIV episode

Within the PHDC, episodes are a term used to describe health conditions, and they can either be acute or chronic. Instead of relying solely on registers or ICD10 classifications, episodes are inferred based on relevant evidence that can be extracted from the available data. HIV is a chronic episode inferred from evidences that include laboratory, pharmacy, diagnosis coding, and observational data. The HIV evidences were compiled by data scientists with clinical domain knowledge in close consultation with clinicians with experience in the services. The evidences have been adjusted to account for the complexities and nuances in the source data. The evidences are under regular revision based on feedback received, but revisions to evidences should undergo a thorough impact review process prior to deployment as a small change to one evidence rule can impact multiple patients. The evidences are under active revision and simplification. Revisions to evidences undergo an impact review process prior to deployment as a small change to one evidence rule can impact multiple patients.

## Episode evidences

On a daily basis, evidences are extracted from the various clinical data structures. Every evidence is combined into a single record per patient per episode that includes key information such as the first and last evidence dates, treatment start dates, facilities at each date point, the list of data sources contributing the evidences, the list of evidences contributing to the episode, an end date (where we have a record of death), and a confidence score.

Every evidence that is used to infer an episode contributes to our confidence in that episode. Additional types of evidence and/or re-occurrence of the same evidence strengthens the episode. The measure of confidence in an episode is cumulative: the higher the total score the more and stronger evidence there is to support the episode. At present, we weight evidences based on four categories:

- High confidence, defining: This evidence is enough on its own to infer an episode. For example, an elevated viral load test is indicative of HIV positive patients. (Score assigned between 0.7 and 1)
- Weak-moderate confidence: This evidence should be cautiously used on its own. For example, some ICD10 diagnosis codes, particularly ones captured prior to discharge, have been shown to be error prone, and in many cases, we prefer to assign a low confidence episode based on an ICD10 code alone. However, if a patient received multiple diagnosis codes in a valid period, the confidence in that episode will increase. (Score ranges <0.25 is considered very weak, 0.25 to 0.5 is considered weak, and 0.5 to 0.7 is considered moderate)
- Supporting evidence: This evidence could not infer an episode on its own but would improve the confidence in an existing episode. For example, dispensing of nausea meds and iron and folate supplements can strengthen a pregnancy episode in that they are evidence of continued care, but is not enough to start the episode on its own. (Score assigned is typically low, such as 0.1, but it could be elevated depending on the evidence.)
- Negating evidence: These evidences do not have a score, but remove an episode if the conditions are met. For example, a negative HIV test within a few days of a positive test, without further evidence of HIV most likely indicate a false positive test and the HIV episode should be removed.

All evidences are combined into one line per episode using a programmatic rollup procedure. For chronic conditions, like HIV, all evidences are combined into one line per patient for their lifetime and episodes are closed only by a death record or a curative evidence if applicable. Individual evidence scores are combined using the following formula to get a value between 0 and 1:

1 - (1-[weight of evidence_1])x(1-[weight of evidence_2])…x(1-[weight of evidence_N])

The confidence score assigned to an episode is a decimal value that ranges from 0-1. As a rule of thumb, scores greater than or equal to 0.7 have at least one high confidence evidence, or more than 5 weak-moderate confidence evidences. Although this can be used for quick filtering, we would suggest that users interpret their confidence in the episode based on the evidence used to infer it. For HIV we suggest that a score of 0.7 be used to identify high confidence episodes. Table 1 details the conditions for each of the evidences currently included in the HIV episode.


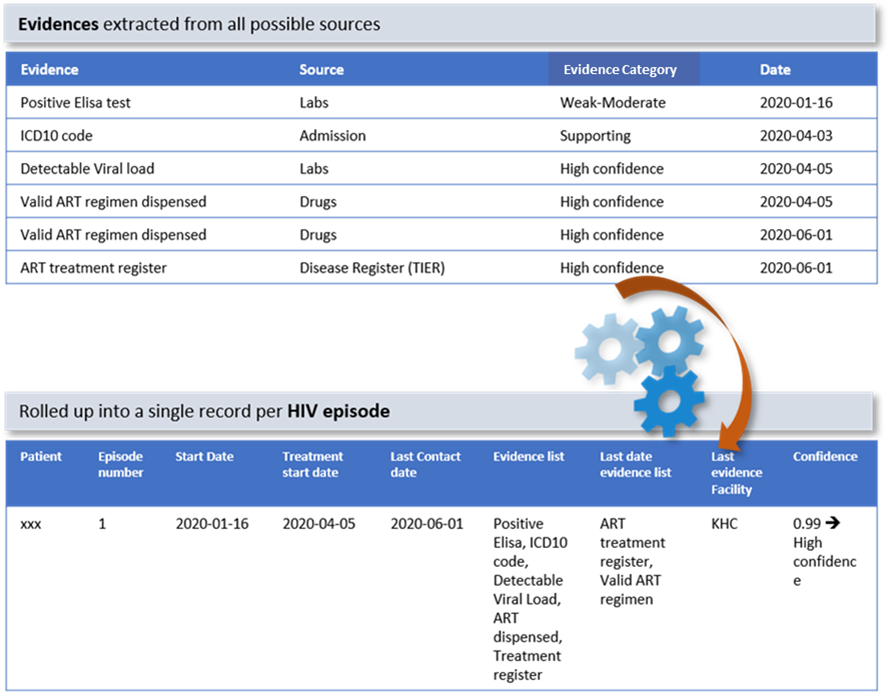


Fig G in S1 Appendix. Depiction of the HIV evidence rollup.

Table A in S1 Appendix. Descriptions of each evidence that contributes to the HIV episode

|  | Event type | Evidence name | Detailed description |
| --- | --- | --- | --- |
| High confidence evidence | | | |
|  | Drug dispensed | ART chronic dispensing | Triple therapy ART dispensed via the CDU. Assumes that CDU will not be used for prophylaxis. Triple therapy is a requirement as ARVs can be used for the treatment of Hepatitis B. |
|  |  | ART treatment register | ART dispensing recorded in an HIV treatment register (PHCIS, PREHMIS, TIER). This assumes that these systems will not be dispensing prophylaxis to registered HIV patients. |
|  | Lab test | ART resistance test | Tests for ART resistance are only conducted on HIV positive individuals. As such, in the absence of any other evidence a test for ART resistance can be indicative of HIV. |
|  |  | Positive Elisa | A positive HIV Elisa test result in all individuals over the age of 2 years old. Elisa tests detect HIV specific antibodies. For breastfeeding infants, the results may be influenced by maternal HIV specific antibodies, and as such in accordance with guidelines, positive Elisa results are only considered for individuals over 2 years of age. |
|  |  | Positive PCR | A positive HIV PCR test result in children under 10 years received via the laboratory system. HIV PCR test results in individuals older than 10 years have been excluded as the guidelines recommend PCR testing for children only. In addition, it is common for PCR results for a child to be recorded on a mother's folder number, therefore to prevent inaccurate classifications an age cutoff of 10 years old was applied. |
|  |  | Positive Rapid Labs | A positive HIV Rapid antibody test result that was received via the laboratory for individuals that are 2 years or older. For breastfeeding infants, the results may be influenced by maternal HIV specific antibodies, and as such in accordance with guidelines, positive Elisa results are only considered for individuals over 2 years of age. |
|  |  | Viral Load Detectable | A HIV Viral load test with an assigned numerical value > 25. 25 was used as this was the lowest level the instrument could detect. All Viral loads that have a result listed as LDL (lower than detectable limit) have a numeric value of 24 assigned to them. A decision was made to separate detectable viral loads from LDL viral loads as in some cases viral load tests were used diagnostically. |
|  | Observations | Confirmatory Positive Rapid Observation | Any confirmatory rapid test positive result for an individual over 2 years of age, that was recorded as an observation into any patient management system that allows for rapid test result capture (e.g. PREHMIS) is used as a high confidence evidence of HIV. |
|  | Activities | Confirmatory Positive Rapid RMR | Positive rapid test results started being scanned and captured for routine monthly reporting in 2022. Any confirmatory positive rapid test for an individual over 2 years of age that was scanned for RMR is used as a high confidence evidence of HIV in accordance with testing guidelines. |
| Weak to Moderate evidence | | | |
|  | Drug dispensed | Triple ARVs 2 months+ acute | Triple ARV therapy dispensed (as components or FDC) to any person over the age of 2 years for two months or more via a source that is not a register source (e.g. not PHCIS/PREHMIS/TIER) and not via a chronic dispensing source (e.g. not CDU). This includes JAC as a source, as ART is not differentiated from other ARV dispensing. |
|  | Lab test | HIV panel CD4 Low | Absolute CD4 counts that have been ordered against the HIV request panel, where the result is less than 350 cells/microlitre. While a low Absolute CD4 count is not in itself indicative of HIV, the fact that it was specifically ordered against the HIV test panel, which is supposed to be for HIV positive patients, and the lack of early rapid testing results, indicates that the clinician who ordered the test was either treating the patient for HIV or suspected HIV infection. As such, these are included as a low confidence evidence. |
|  |  | Viral Load LDL | An HIV viral load lower than detectable limit, can be an evidence of ongoing HIV care. Although an LDL viral load is not in itself indicative of HIV, this test is typically only ordered for HIV positive patients unless the clinician is trying to test for HIV indirectly. The existence of the test, indicates that the clinician who ordered the test was either treating the patient for HIV or suspected HIV infection. As such, these are included as a low confidence evidence. |
|  | Procedure or diagnosis coding | ICD10 HIV Positive | Diagnosis codes recorded into Clinicom, ECCR or HECTIS indicate that the patient is HIV Positive. Diagnosis codes are prone to capture errors, though this is improving over time. To ensure no patient is lost, weak evidences are created based on diagnosis codes in the absence of laboratory or HIV treatment register evidence. |
|  | Observations | HIV positive status recorded | HIV positive status is captured into HIV treatment registers, TB treatment registers, as well as discharge summary and other electronic medical record forms. These are stored in an observations data structure within the PHDC and used as an evidence. |
| Supporting evidence | | | |
|  | Drug dispensed | ARVs for infants | For infants under 24 months with any high confidence evidence of HIV (e.g. PCR positive or Treatment register entry), any ARVs dispensed are used as evidence of treatment, except for tenofovir based regimens, which are not used for infants. Treatment is used as a supporting evidence as ARVs may be given as prophylaxis in infancy. |
|  |  | Triple ARVs<2month acute | Triple ARV therapy dispensed (as components or FDC) to any person over the age of 2 years for less than 2 months via a source that is not a register source (e.g. not PHCIS/PREHMIS/TIER) and not via a chronic dispensing source (e.g. not CDU). This includes JAC as a source, as ART is not differentiated from other ARV dispensing. |
|  | Lab test | HIV panel CD4 High | Absolute CD4 counts that have been ordered against the HIV request panel, where the result is greater than or equal to 350 cells/microlitre. While a high Absolute CD4 count is not indicative of HIV, it can be used as a supporting evidence to show ongoing care for HIV. |
| Negating evidence | | | |
|  | Lab test | Negative Elisa | Negative HIV Elisa test for an individual over 2 years of age, that was sent via the laboratory can be used to negate weak-to-moderate evidences of HIV. ART dispensed via chronic dispensing can be negated as while triple therapy chronic ARVs should only be used for HIV, the evidence is not diagnostic and occasional dispensing that may be PrEP has been observed. |
|  |  | Negative PCR | Negative HIV PCR test for an individual under 10 years of age can be used to negate weak-to-moderate evidences as well as Positive PCRs (due to birth PCRs being influenced by maternal HIV), and any ART dispensed via chronic dispensing. ART dispensed via chronic dispensing can be negated as while triple therapy chronic ARVs should only be used for HIV, the evidence is not diagnostic and occasional dispensing that may be PrEP has been observed. |
|  |  | Negative Rapid Labs | Negative rapid test for an individual over 2 years of age, that was sent via the laboratory can be used to negate weak-to-moderate evidences of HIV. ART dispensed via chronic dispensing can be negated as while triple therapy chronic ARVs should only be used for HIV, the evidence is not diagnostic and occasional dispensing that may be PrEP has been observed. |
|  | Observations | Negative Rapid Observation | Negative screening rapid test for an individual over 2 years of age, that was recorded as an observation into any patient management system that allows for rapid test result capture (e.g PREHMIS) can be used to negate weak-to-moderate evidences of HIV. ART dispensed via chronic dispensing can be negated as while triple therapy chronic ARVs should only be used for HIV, the evidence is not diagnostic and occasional dispensing that may be PrEP has been observed. |
|  | Activities | Negative Rapid RMR | Negative screening rapid test for an individual over 2 years of age, that was scanned or captured for routine monthly reporting can be used to negate weak-to-moderate evidences of HIV. ART dispensed via chronic dispensing can be negated as while triple therapy chronic ARVs should only be used for HIV, the evidence is not diagnostic and occasional dispensing that may be PrEP has been observed. |
|  | Activities | Patient on PrEP | If there is indication in the RMR data that the patient has initiated on PrEP, then the patient has to have screened Negative for HIV. This can be used to negate any very weak confidence evidences. |

Contribution of evidences to episode ascertainment

To summarise the contribution of evidences to the episode ascertainment we have developed an internal dashboard that can be accessed by individuals who have access to the clinical and reporting tools made available by the PHDC.

<https://app.powerbi.com/links/_Ss35U7mgb?ctid=ae74bf7f-cfc3-4760-a1fe-0731afaa5502&pbi_source=linkShare&bookmarkGuid=8e1d545c-0cfd-4e59-b1e9-41c28ee03cce>

# The HIV cascade

There is a requirement to enable the PHDC to report cross-sectionally on the status of each person known to be living with HIV in the WC at any point in time, and longitudinally on the changes of status of each person over time. To do this, the most recent information is required at a person-level at each and every potential report date. To enable this, two core data tables are required. Firstly, a table that contains one row per patient with any information that does not change, which we called the “baseline” table. And a second table that contains a row per patient per date on which any information may have changed, which we called the “visits” table.

## The baseline table

The baseline table contains any information only occurs once, e.g. date of birth. The full list of columns is provided in Table 2.

Table B in S1 Appendix. Tables and columns in the baseline and visits HIV cascade intermediate tables

| **Table** | **Columns** | **Data type** | **Label in branch logic** |
| --- | --- | --- | --- |
| baseline | person_id  location_id  date_of_birth  sex  hiv_dx_date  art_start_date  art_start_cd4  date_of_death | integer integer date integer date date integer date | art  death |
| visits | person_id  visit_date  facility_id  last_hiv_test_date  last_art_dispensed_date  last_art_duration  last_regimen_id  last_art_in_hand_date  last_vl_date  last_vl_result  last_cd4_date  last_cd4_result  last_return_to_care_date  last_rpcs_date  last_tb_start_date last_tb_end_date  last_pregnancy_start_date  last_pregnancy_end_date | integer date integer date date integer integer date date integer date integer date date | visit  test    laih vl copies cd4 |
| cascade | person_id  visit_date  last_facility_id  last_test_date  last_rtc_date  last_rpcs_id  last_art_date  last_art_in_hand_date  regimen_id  months_since_last_seen  last_cd4_date  last_cd4_result  last_start_cd4  last_vl_date  last_vl_result  cascade_status_id  active_tb  active_pregnancy  report_date  status_lag4_id  status_lag2_id  status_lag1_id  status_duration_months | Integer date integer date date integer  date date integer integer date integer integer date integer integer integer integer date integer integer integer integer |  |

## The visits table

The visits table starts with the encounters table described above. Every time a patient interacts with the health services, an encounter is inferred. Using encounters as a “spine”, the most recent information about each patient at each encounter date is calculated. The most important information is when last the patient received ART, how many days of ART they received and, combining these, what is the date on which they may run out of ART, which would represent a potential treatment interruption is additional ART is not collected. Markers of treatment success or failure, such as the most recent VL, are also carried. This can be thought of as “carrying” the latest information forward over consecutive rows through time, as depicted in Figure 8.


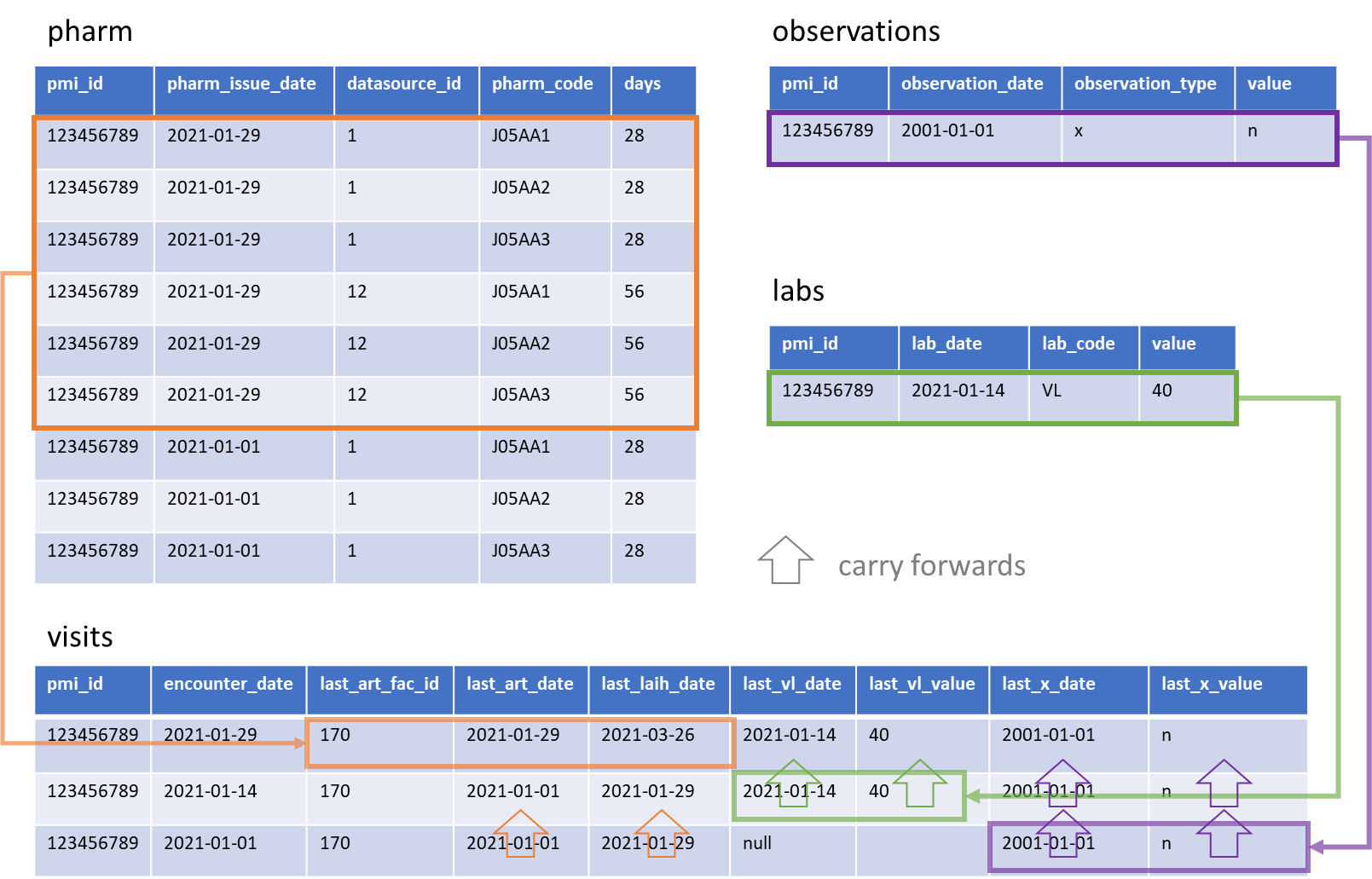


Fig H in S1 Appendix. Illustration of “carrying” forwards in visits table.

## The branch logic

A reporting calendar was populated with report dates based on three parameters: a) a report end date, b) a reporting duration, and c) a reporting period between successive report dates. For example, the calendar could be populated up until the end of the most recent quarter, for five years, with a quarterly reporting period, which would lead to 20 report dates.

We developed branch logic to allocate each person a cascade status at each report date (Figure 9). Cascade statuses were mutually exclusive (each person could have only one status at each report date) and mutually exhaustive (every person had one status at each report date after their HIV episode start date).


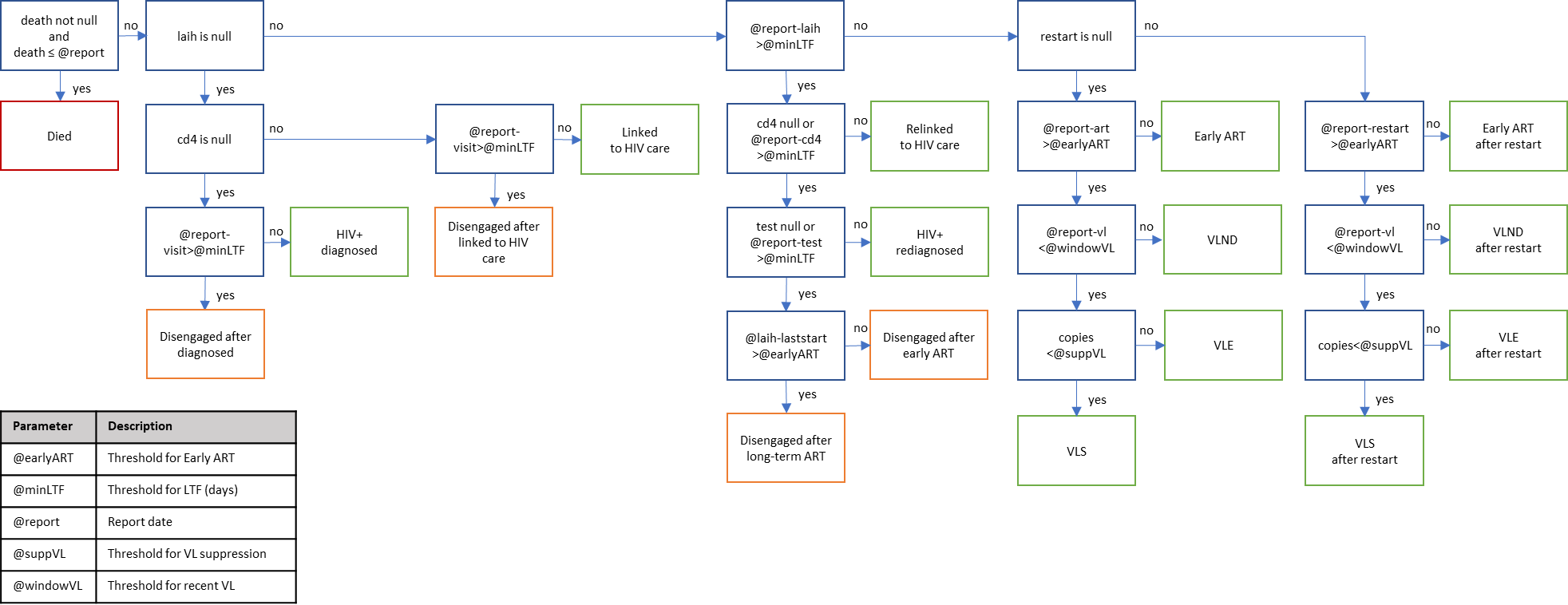


Fig I in S1 Appendix. The cascade branch logic.

# References

1. Harklerode, R., et al., *Feasibility of Establishing HIV Case-Based Surveillance to Measure Progress Along the Health Sector Cascade: Situational Assessments in Tanzania, South Africa, and Kenya.* JMIR Public Health Surveill, 2017. **3**(3): p. e44.

2. Holmes, J.R., et al., *Status of HIV Case-Based Surveillance Implementation - 39 U.S. PEPFAR-Supported Countries, May-July 2019.* MMWR Morb Mortal Wkly Rep, 2019. **68**(47): p. 1089-1095.

3. Coetzee, D., et al., *Outcomes after two years of providing antiretroviral treatment in Khayelitsha, South Africa.* AIDS, 2004. **18**(6): p. 887-95.

4. Kaplan, S.R., et al., *Contemporary disengagement from antiretroviral therapy in Khayelitsha, South Africa: A cohort study.* PLoS Med, 2017. **14**(11): p. e1002407.

5. Stinson, K., et al., *Cohort Profile: The Khayelitsha antiretroviral programme, Cape Town, South Africa.* Int J Epidemiol, 2017. **46**(2): p. e21.

6. *2019 ART Clinical Guidelines for the Management of HIV in Adults, Pregnancy, Adolescents, Children, Infants and Neonates*. Updated March 2020, Republic of South Africa National Department of Health: Pretoria, South Africa.

7. Luque-Fernandez, M.A., et al., *Effectiveness of patient adherence groups as a model of care for stable patients on antiretroviral therapy in Khayelitsha, Cape Town, South Africa.* PLoS ONE, 2013. **8**(2): p. e56088.

8. Tsondai, P.R., et al., *High rates of retention and viral suppression in the scale-up of antiretroviral therapy adherence clubs in Cape Town, South Africa.* J Int AIDS Soc, 2017. **20**(Suppl 4): p. 21649.

9. Abdullah, M.F., et al., *Public health lessons from a pilot programme to reduce mother-to-child transmission of HIV-1 in Khayelitsha.* S.Afr.Med.J., 2001. **91**(7): p. 579-583.

10. Osler, M., et al., *A three-tier framework for monitoring antiretroviral therapy in high HIV burden settings.* J Int AIDS Soc, 2014. **17**: p. 18908.

11. Beck, E.J., et al., *Developing and implementing national health identifiers in resource limited countries: why, what, who, when and how?* Glob Health Action, 2018. **11**(1): p. 1440782.

12. Boulle, A., et al., *Data Centre Profile: The Provincial Health Data Centre of the Western Cape Province, South Africa.* Int J Popul Data Sci, 2019. **4**(2): p. 1143.

13. *2021 Health Normative Standards Framework for Digital Health Interoperability in South Africa*. 2022, Repubic of South Africa Department of Health: Pretoria.

# List of legends

Fig A in S1 Appendix. Data transfer and integration within the Western Cape Provincial Health Data Centre
Fig B in S1 Appendix. Theory of change for digital support to a responsive patient-centred health system
Fig C in S1 Appendix. Integration of source data into the Western Cape Provincial Health Data Centre data structures
Fig D in S1 Appendix. Linking source data to the Patient Master Index
Fig E in S1 Appendix. Deduplicating records within the Patient Master Index
Fig F in S1 Appendix. Structure of the PHCIS data received by the PHDC
Fig G in S1 Appendix. Depiction of the HIV evidence rollup
Table A in S1 Appendix. Descriptions of each evidence that contributes to the HIV episode
Table B in S1 Appendix. Tables and columns in the baseline and visits HIV cascade intermediate tables
Fig H in S1 Appendix. Illustration of “carrying forwards” in the visits table
Fig I in S1 Appendix. The cascade status branch logic

**Contact**

Email: Provincial Health Data Centre: phdc_pgwc@westerncape.gov.za

**Department of Health and Wellness**

**Directorate: Health Intelligence**

[**www.westerncape.gov.za**](http://www.westerncape.gov.za)
